# Scale‐Up of Solvent‐Free, Mechanochemical Precursor Synthesis for Nanoporous Carbon Materials via Extrusion

**DOI:** 10.1002/cssc.202200651

**Published:** 2022-06-28

**Authors:** Tilo Rensch, Viviene Chantrain, Miriam Sander, Sven Grätz, Lars Borchardt

**Affiliations:** ^1^ Department of Inorganic Chemistry Ruhr-Universität Bochum Universitätsstrasse 150 44801 Bochum Germany

**Keywords:** extrusion, mechanochemistry, nitrogen-doped carbon, nanomaterials, sustainability

## Abstract

The mechanochemical synthesis of nitrogen‐rich nanoporous carbon materials has been scaled up using an extruder. Lignin, urea, and K_2_CO_3_ were extruded under heat and pressure to yield nanoporous carbons with up to 3500 m^2^ g^−1^ specific surface area after pyrolysis. The route was further broadened by applying different nitrogen sources as well as sawdust as a low‐cost renewable feedstock to receive carbons with a C/N ratio of up to 15 depending on nitrogen source and extrusion parameters. The texture of obtained carbons was investigated by scanning electron microscopy as well as argon and nitrogen physisorption, while the chemical structure was analyzed by X‐ray photoelectron spectroscopy. The received carbon was tested as a supercapacitor electrode, showing comparable performance to similar ball‐mill‐synthesized materials. Lastly, the space‐time yield was applied to justify the use of a continuous reactor versus the ball mill.

## Graphical abstract

**Mechanosynthesis**: The synthesis of nitrogen‐rich nanoporous carbon materials is scaled up using an extruder as a mechanochemical reactor. Lignin, urea, and K_2_CO_3_ are extruded under heat to yield nanoporous carbons with up to 3500 m^2^ g^−1^ specific surface area after pyrolysis. Biuret, melamine, and sawdust are explored as renewable feedstock to enable N‐rich nanoporous carbons as competitive materials to traditional activated carbons.

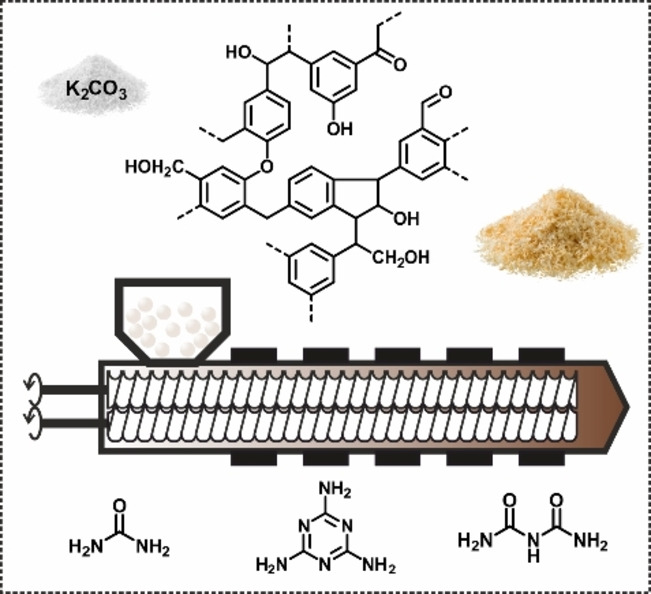

## Introduction

Due to changes in climate, occurring natural disasters, and shortage of oil‐based fuels the matter of environmentally friendly processes and sustainable energy has become urgent. Nanoporous carbons (NC) play a key role in air and water purification to scrub toxins and greenhouse gases from the environment.^[1]^ They are suitable as catalysts or catalyst supports, for gas sorption and separation, and in energy storage devices like supercapacitors, fuel cells, or batteries.[^2^, ^3^, ^4^]

Most industrially relevant syntheses rely on either the physical or chemical activation of organic compounds such as coconut shells, lignin, or rice husks.[^3^, ^5^] Due to lower control over the surface, pore structure, and impurities, their performance in most applications (e. g., electrochemical energy storage) is lower than that of tailored carbons obtained by templating, sol–gel chars, or other advanced synthesis techniques.^[6]^ To overcome these limitations there is a great need for tailoring the surface properties of NC by doping the carbon precursor with heteroatoms, adding hierarchical pore architecture, or controlling the carbonization degree to increase their desired performances.[^4^, ^7^, ^8^, ^9^, ^10^] One strategy that gained great importance over the last years is the incorporation of nitrogen into the carbon structure. Nitrogen‐doping was shown to be beneficial for conductivity, capacity in storage devices, and electrolyte wettability.[^7^, ^11^] Nitrogen is incorporated either in the final carbon or already into the precursor.[^12^, ^13^] Most of the existing protocols rely on multiple reaction steps. Recently, nitrogen‐doped carbons with specific surface areas (SSA) of up to 1500 m^2^ g^−1^ and up to 25 % nitrogen content were obtained by pyrolysis of urea‐doped ZIF‐8.^[14]^ Other approaches involving metal–organic frameworks and further nanostructures could also reach high SSA up to 1731 m^2^ g^−1^ and pore volumes up to 1.68 cm^3^ g^−1^.^[15]^ Activating natural materials or waste plastics can yield nitrogen‐rich NC with SSA between 2342 and 1690 m^2^ g^−1^, respectively.^[16]^ Typically, off‐the‐shelf NCs reach specific capacitances upwards of 100 F g^−1^, while highly‐tailored NCs can reach specific capacitances up to 330 F g^−1^.^[17]^ However, these approaches do not only produce enormous amounts of waste but often take a long time as wet chemical routes rely on solvents, while gas‐phase syntheses use nitrogen‐containing gases. Besides these classical methods, our group introduced a synthesis concept based on mechanochemistry.[^9^, ^12^] Renewable carbon sources such as lignin, cellulose, or wood waste are milled together with the nitrogen source (urea) and an activation agent (K_2_CO_3_) inside a ball mill. Both K_2_CO_3_ and urea are cheap and abundantly available; moreover, the by‐products can serve as an “in‐situ electrolyte” when dissolved, which makes subsequent reprocessing obsolete.[^8^, ^18^]

Mechanochemical approaches are solid‐state reactions, thus making the solvent (the largest contributor to chemical waste) obsolete, rendering this concept highly sustainable. Mechanical energy provides the necessary activation energy for the chemical reaction, which proceeds quickly and results in high yields.^[19]^ It was shown that this method is a powerful tool for the synthesis of porous carbonaceous materials.[^20^, ^21^] At the moment the most prominent way to introduce the mechanical energy into the reactants is by grinding in a ball mill.^[22]^ They have exhibited tremendous potential in all fields of chemistry and can even increase electrochemical performance, but the scalability of ball mills is limited.[^21^, ^23^] Also, conditions are difficult to control and monitor as the closed grinding jar causes a black box character.^[24]^ Another disadvantage of ball mills is their batch process, making it unfavorable for industrial application. A possible continuous alternative with high process control is extrusion, which is already established in the food and polymer industries. Twin‐screw extrusion (TSE) has proven to reproduce reactions performed in ball mills and was applied to produce materials like metal–organic frameworks.^[25]^

**Figure 1:**
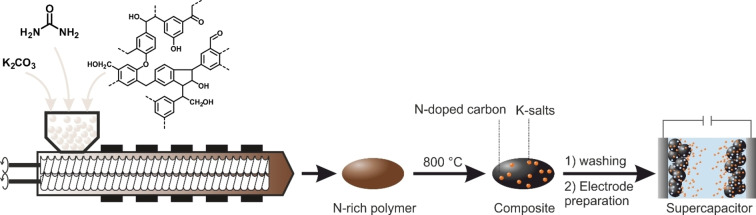
Schematic drawing of developed twin‐screw extrusion process for synthesis of N‐doped carbon composites with application in supercapacitors.

Herein, we introduce a continuous process for the preparation of a nitrogen‐rich precursor polymer in the extruder. Subsequently, the polymer undergoes carbonization to N‐doped porous NC (Figure 1). To prove the quality of the obtained product, we compare it against previously reported materials and apply a range of different substrates as well as process parameters. Furthermore, the resulting carbon was tested as a supercapacitor to give an idea for further applicability.

## Results and Discussion

In general, reactivity in extruders is caused by two sources. On the one hand, the temperature commonly plays a significant role, and, on the other hand, so does the shear force imbued into the reaction mixture. These shear forces are promoted by high‐pressure areas along the barrel and can be facilitated by kneading zones in the extrusion screws. Thus, we opted for a screw design containing two separate kneading zones (Figure S1). For the reaction mixture, a similar approach to that by Schneidermann et al. was chosen with a 1 : 1 : 1 mixture of lignin, urea, and K_2_CO_3_, denoted in the following as LUK.^[9]^

**Figure 2:**
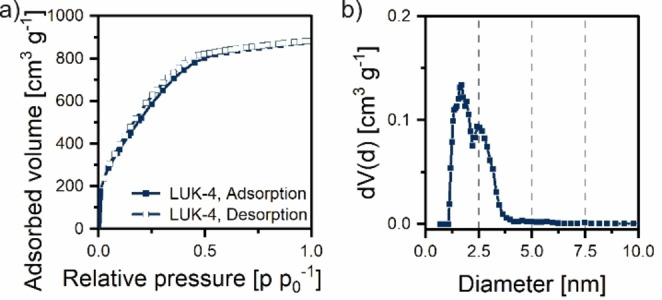
(a) Ar physisorption isotherm of sample LUK‐4 showing type I b behavior. (b) Incremental pore size distribution calculated from Ar isotherm for sample LUK‐4.

In a typical extrusion, these raw materials were hand‐fed into the extruder at a set rate of 1 g min^−1^ and a rotational speed of 55 rpm at 100 °C (Table 1). Pyrolysis yields an activated carbon with a carbon yield of 13.1 % (Table S1). Similar to the ball mill process, dense polymer particles are obtained as shown in scanning electron microscopy (SEM) images (Figure S2a,d). In contrast to the particles produced by ball milling, there are also smaller‐sized particles agglomerated on the surface of larger particles. Electron‐dispersive X‐ray spectroscopy (EDS) reveals that the smaller‐sized particles contain a large amount of nitrogen (Figure S3). These are most likely unreacted particles of urea that were not fully incorporated into the lignin matrix. After pyrolysis, particulates with homogeneous nitrogen distribution and visible macroporosity remain (Figure S2b, e). EDS also reveals the presence of not only the expected elements C, N, O, and K but also of sodium and sulfur, remnants from lignin processing (Figure S4). However, washing these samples (Figure S5) removes these impurities. X‐ray photoelectron spectroscopy (XPS) of all stages confirms that these impurities make up only 1.5 at % of the whole sample after extrusion, increase to 6.2 at % for the composite, and are below the detection limit for the final carbon product (Table S4). Furthermore, the final carbon has a C/N ratio of 32. Since SEM images show unreacted urea on lignin particles, we employed IR spectroscopy to analyze the freshly extruded polymer (Figure S6, 100 °C, bright red). New signals around 2200 and 1030 cm^−1^ appear. The vibration around 2200 cm^−1^ belongs to nitrile or isocyanate groups (decomposition products of urea). The second vibration around 1030 cm^−1^ is present due to oxidation of sulfur impurities. X‐ray diffraction (XRD) of the composite after pyrolysis show KCNO as the remaining salt species (Figure S39). This stems from the observed unreacted urea that decomposes during pyrolysis but fully dissolves during HCl washing, leaving an activated carbon with an SSA of 1955 m^2^ g^−1^ (Ar, Table S5), respectively 3326 m^2^ g^−1^ (N_2_, Table S6, entry 4) (Figure 2a). The pore structure consists of mostly micropores (Figure 2b) with some small mesopores up to 4 nm in diameter leading to a total pore volume of 1.121 cm^3^ g^−1^ (Ar, Table S5), and 1.783 cm^3^ g^−1^ (N_2_, Table S6 entry 4), respectively. The discrepancy between physisorption using Ar and N_2_ is an established phenomenon on polar surfaces and is attributable to the quadrupole moment of the N_2_ molecule. In this regard, the Ar value is a closer representation of the real surface; however, for sake of comparability to previously published results, N_2_ values will be mentioned and compared hereafter.

**Table 1:** Optimization of extrusion parameters concerning carbon yield, nitrogen content (C/N), and SSA calculated from N_2_ physisorption.

Entry	ID	ω^[a]^ [rpm]	*ϑ* ^[b]^ [°C]	*f* ^[c]^ [g min^−1^]	C‐yield [%]	C/N	SSA [m^2^ g^−1^]
1	LUK‐1	55	25	1	3.9	41.0	3211
2	LUK‐2	55	50	1	6.9	91.6	3380
3	LUK‐3	55	80	1	10.4	15.0	2520
4	LUK‐4	55	100	1	13.1	32.5	3326
5	LUK‐5	55	120	1	15.5	72.3	2762
6	LUK‐6	45	100	1	11.0	53.8	2804
7	LUK‐7	75	100	1	11.5	37.7	2943
8	LUK‐8	95	100	1	14.8	35.5	2773
9	LUK‐9	150	100	1	10.5	76.3	3037
10	LUK‐10	55	100	1.5	17.2	70.9	3126
11	LUK‐11	55	100	2	12.5	37.8	3857
12	LUK‐12	55	100	5	2.0	17.3	2739
13	LMK‐1	55	100	1	3.7	17.0	2934
14	LMK‐2	55	140	1	4.2	19.9	3165
15	LMK‐3	55	300	1	6.4	17.3	3316
16	LBK‐1	55	100	1	6.2	56.5	3384
17	LBK‐2	55	180	1	14.1	142.3	2962
18	LUK‐BM^[d]^	55	100	1	5.2	15.1	2575
19	LUK‐CS^[e]^	55	100	1	12.2	69.4	2393
20	LK^[f]^	55	100	1	21.2	140.9	584
21	WUK^[g]^	55	100	1	8.5	29.0	2581

[a] *ω*=screw speed. [b] *ϑ*=barrel temperature. [c] *f*=feed rate. [d] Sample produced by ball milling. [e] Sample produced by extrusion without kneading elements. [f] Reaction mixture without urea. [g] Lignin replaced by wood waste.

### Optimization of extrusion conditions

Before tuning the parameters, we analyzed the physical reaction mixture and the reaction mixture after extrusion using differential scanning calorimetry (Figures S37 and S38). While both graphs show very similar shapes, the onset temperatures for signals in the extruded mixture lie roughly 20 K below the onset temperature for the untreated mixture. This is an effect of the energy introduced during extrusion. Since potassium carbonate decomposes far beyond the temperatures reached during extrusion and urea only starts decomposing above 130 °C, the signals at 80 °C in the physical mixture and 60 °C in the extruded mixture must stem from reactions of lignin and urea.^[26]^ As these signals are still present after extrusion, harsher reaction conditions must be applied to fully exploit the potential of the reaction mixture. This compares well to the images obtained in SEM since unreacted urea is seen after extrusion.

**Figure 3:**
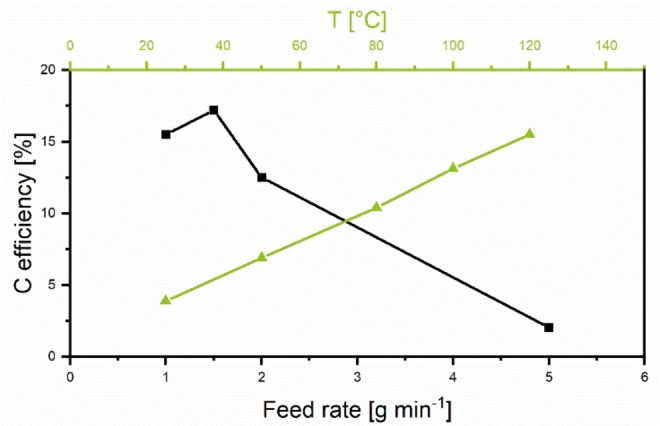
Carbon efficiency as a function of extrusion temperature at a feed of 1 g min^−1^ (green, LUK‐1‐5) and as a function of feed rate at 100 °C (black, LUK‐4 and LUK‐10‐12).

Using this knowledge, we opted to start with temperature variations as a crucial parameter. As temperature does not only impact the reaction rate, but also the rheology of the mixture inside the barrel and thus the shear forces exerted onto the reaction mixture it is important to optimize it before changing the filling grades of the extruder (Table 1). While doing so, we encountered 120 °C as the upper limit for a feasible extrusion temperature as any further increase in temperature led to a change in rheology from a paste‐like mixture into a consistency that blocked the screws. This is most likely due to the removal of water from the reaction mixture and the onsetting decomposition of urea above 133 °C.^[26]^ Lower temperatures produce samples with lower carbon yield overall (Figure 3, green), while the C/N ratio varies between 15 and 91. Previously, it was reported that only K_2_CO_3_ remains in the composite material after pyrolysis. However, we find species ranging from K_2_O, KCN, KCNO, and K_2_CO_3_ in our samples after pyrolysis, implying partial polymer formation and hindered diffusion of pyrolysis gases during activation (Figures S39–S43). When varying screw speeds in the extrusion process no significant impact on carbon efficiency can be noted, while increasing the feed rate hurts the carbon (C)‐efficiency (Figure 3, black). The C‐efficiency is calculated from the mass remaining after pyrolysis under the assumption that every carbon species except K_2_CO_3_ can be directly transformed into carbon. Thus, the C‐efficiency measures the effective polymerization and distribution of K_2_CO_3_ inside the polymer matrix, which results in homogeneous activation. At higher feed rates the C‐efficiency drops, resulting in lower C/N ratios as carbonaceous species are gasified before tightly bound nitrogen species. As extrusion is a continuous process, not only yields have to be considered but also space‐time yields (STY), where throughput is considered (Table S5). STY for this process range from 1657 kg m^−3^ d^−1^ for a low feed at low temperatures up to 8614 kg m^−3^ d^−1^ for LUK‐10 with a direct correlation for screw speed, feed rate, and C‐efficiency as to be expected (Table S5). Taking SSA, C/N ratios, C‐efficiency, and STY into consideration, sample LUK‐4 was elected for reference conditions. To prove the impact of mechanical force on the reaction, screws only containing conveying elements were utilized (Table S3, entry 3). Even though a respectable C‐yield was reached, the resulting carbon had significantly lower SSA compared to the sample with kneading elements.

### Alternative feedstock

To display the capabilities of the developed process, we opted to vary the N‐source. Here, biuret as the dimer of urea, as well as the trimer melamine were used (Table S2). For these reactants, it was possible to further increase the temperature close to their respective decomposition temperatures. At 100 °C both alternative N‐sources result in comparable C/N ratios and SSA as observed before. However, the C‐yield is significantly lower at below 7 %. Increasing the temperature to 300 °C still yields a similar product, making melamine a potential precursor for nitrogen‐rich carbon of this type. In contrast to urea, the samples with melamine and biuret did not form a viscous mass inside the extruder, leading to less dispersion of potassium carbonate in the polymer and a more inhomogeneous activation, leading to lower C‐yield at the same level of activation. Since all synthesized carbons to this point had high SSA of between 2000 and 3500 m^2^ g^−1^, we wanted to synthesize a carbon without urea addition (Table S3, entry 4). Even though the C‐yield for this product was the highest of the entire set of carbons, the SSA lagged far behind the other materials at only 500 m^2^ g^−1^, showing the significance of a nitrogen source on this reaction pathway. Reason for this rather low surface area is the change in rheology to a more solid mixture and thus, worse dispersion of K_2_CO_3_ throughout the sample. Additionally, we also exchanged lignin as a feedstock material for raw wood waste (Table S3, entry 5). This led also to an exceptional material with high N content of 3.0 %, high SSA of 2581 m^2^ g^−1^, and moderate C‐yield of 8.5 % while still maintaining a comparable STY of 3551 kg m^−3^ d^−1^.

### Electrochemical application

**Figure 4:**
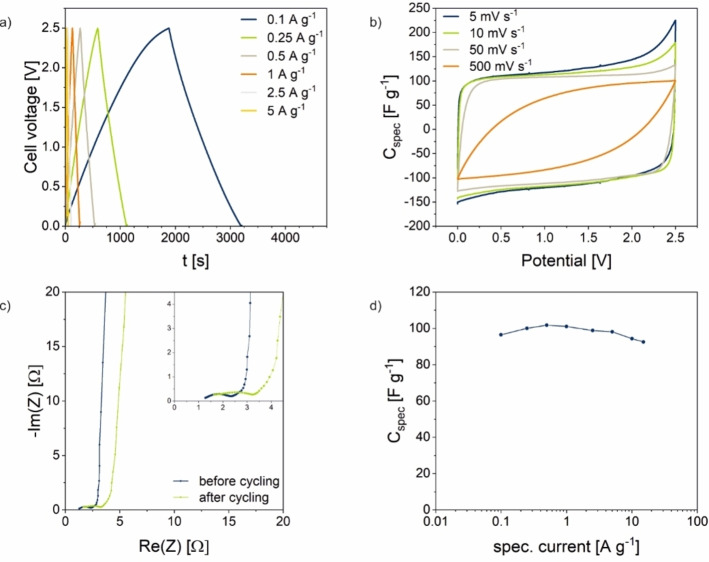
Electrochemical characterization of LUK‐4 in 1 m TEABF_4_/AcN. (a) Galvanostatic charge–discharge plots at varying current densities. (b) CVs at varying scan rates. (c) Nyquist plots before and after cycling. (d) Specific capacitance at different specific currents.

To present its electrochemical performance, the NC LUK‐4 was applied in a supercapacitor with a conventional electrolyte (1 m Tetraethylammonium tetrafluoroborate (TEABF_4_)/AcN). The cyclic voltammograms (CVs) with their rectangular shape as well as charge–discharge measurements (Figure 4a, b) show a typical capacitor‐like behavior, However, slight deviations from the ideal shape are evident, which may be caused by the heterogeneous character of the natural feedstock material. Nitrogen is known to produce surface functionalities associated with redox activity. This is indicated by the CVs, whereby the redox activity in nitrogen‐doped carbons typically occurs at low scanning rates. Accordingly, impedance spectroscopy before and after cyclic voltammetry shows a low system resistance and steep slope for high frequencies (Figure 4c). The increase in resistance during cycling is most likely caused by the observed redox activity coupled with the narrow pore structure, which is disadvantageous for ion diffusion. Energy storage performance was quantified at different specific currents to reveal its optimum at a current density of 1 A g^−1^ with a specific capacitance of 101 F g^−1^ (Figure 4d). Moreover, the presented system shows remarkable stability in capacitance with an increasing current rate.

### Comparison of ball milling vs. extrusion

**Figure 5:**
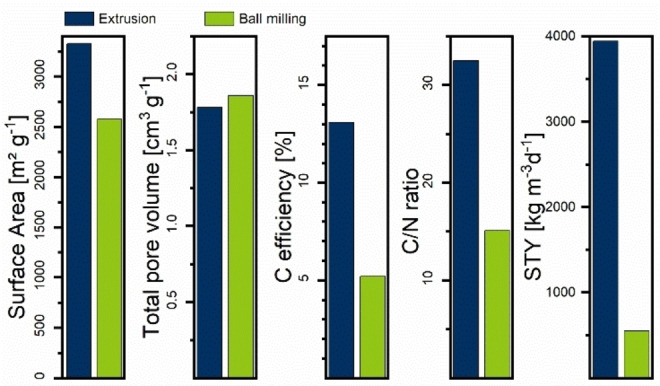
Comparison between LUK sample prepared by extrusion (blue) and ball milling (green).

Lastly, we prepared a sample via ball milling according to Schneidermann et al.,[^9^, ^12^] whereby the same analyses as described before were performed (Figure 5). The extruded sample has a slightly higher SSA while having comparable pore volume. Comparing the pore structure calculated with non‐linear density functional theory (NLDFT, Figure S53) shows fewer micropores for the ball‐milled sample while having an extended network of mesopores. Even though retention time in the extruder is significantly lower than the milling time and the mechanical forces most likely lower, the increase in temperature leads to comparable impregnation of K_2_CO_3_ into the polymer network. The C‐efficiency for the extruded sample is significantly higher, while the C/N ratio is lower for the ball‐milled sample. Ball milling probably has the edge here because high impact forces lead to better incorporation of urea into the lignin network, while extrusion depends on wettability and miscibility in a viscous phase. SEM images (Figure S2a) and EDS measurements (Figure S3) show that urea was indeed not fully incorporated into the polymer network. A higher C‐efficiency and extrusion as a continuous process lead to a roughly sevenfold higher STY at 3941 kg m^−3^ d^−1^ for the extruded sample. The common ball mill approach shows similar electrochemical performance (Figure S54) with a slightly higher capacitance than the extruded material because it was activated to a lower C‐efficiency, resulting in higher relative nitrogen content and a more hierarchical pore system, which promotes charge storage.

## Conclusion

We set out to find a sustainable synthesis pathway leading to nitrogen‐rich precursor polymers for nanoporous carbons (NCs). Herein, we established a scalable synthesis route using extrusion to produce nanoporous carbons with a specific surface area of 3326 m^2^ g^−1^, a pore volume of 1.783 cm^3^ g^−1^, and a C/N ratio of up to 15 while retaining a high C‐yield of typically over 10 %. These results are comparable to previously reported ball milling procedures. We tested the electrochemical performance of the NCs in supercapacitors, which resulted in a good specific capacitance of 76 F g^−1^. Furthermore, we expanded the synthesis route to different nitrogen sources and even wood waste as lignin replacement. The resulting products show similar quality compared to the materials generated from more refined feedstock, thus allowing great process flexibility and establishing a new route for potential industrial application that enables competition with traditional, low‐cost activated carbons.

## Experimental Section

The used extruder is the ZE 12 HMI twin‐screw extruder from Three‐Tec with a 40 *L : D* barrel and two‐coursed, parallel, corotating, and partially combing twin screws. The reaction mixture was premixed by hand and consisted of a 1 : 1 : 1 mixture of the carbon source (lignin=L or powdered wood waste=W), the nitrogen source (urea=U, melamin=M, or biotin=B), and the salt (potassium carbonate=K). The mixture was then fed by hand into the extruder. A steady feed was ensured by monitoring the torque. When torque and temperature were steady, a sample was taken over several minutes to minimize feed variations. The screw configuration was used as shown in Figure S1 and consisted of three conveying zones and two kneading zones. The free volume of the extruder barrel is 46.95 cm^3^. The STY is a chemical engineering parameter to measure efficiency, and it is the amount of product, per reactor volume, per day [kg m^−3^ day^−1^]. STY is calculated using Equation 1:

(1) $$STY=\frac{yield}{day\;\times \;free\;reactor\;volume}$$

The conditions were varied in temperature (LUK=25, 50, 80, 100, 120, 140 °C; LMK=100, 140, 300 °C; LBK=100, 180 °C; WUK=100 °C), rotational speed (LUK=45, 55, 75, 95, 150 rpm), and feed (1, 1.5, 2, and 5 g min^−1^). The chosen standard conditions were 100 °C and 55 rpm at a feed of 1 g min^−1^.

After the extrusion, the samples were pyrolyzed in the Nabertherm Compact Tube Furnace under nitrogen. The used program consisted of heating up to 60 °C at a rate of 150 °C h^−1^ and holding this temperature for 1 h. Then the furnace was heated up to 800 °C at 150 °C h^−1^ and held at that for 2 h. After slowly cooling down to room temperature 0.5 g of the sample was washed with 400 mL half‐concentrated HCl and distilled water until the filtrate was pH‐neutral and then dried at 80 °C. Carbon efficiency was calculated by Equation 2:

(2) $$C-\mathrm{efficiency}=\frac{m_{carbon}}{m_{lignin}+m_{urea}}$$

The solid samples were analyzed by the IR‐Spirit with QATR−S from Shimadzu in the range from 400 to 4000 cm^−1^. The recorded spectra were then normalized. The PXRD spectra were measured on an STOE STADI P with an MYTHEN 1 K detector. Here, the Cu K_α_ radiation was generated at 40 kV and 30 mA and filtered through a Ga monochromator. Physisorption was measured using a Quantachrome Quadrasorb Evo/SI after the samples were activated in the Flovac Degasser (also Quantachrome) for at least 3 h at 150 °C. The adsorption and desorption isotherms were recorded, and the specific surface area was determined by the Brunauer–Emmet–Teller (BET) method. The pore radius distribution was calculated by the DFT method.

The elemental compositions of the washed samples were measured with the Nexsa G2 X‐ray Photoelectron Spectrometer by Thermo Fisher Scientific with ion scattering spectroscopy (ISS), UV photoelectron spectroscopy (UPS), and reflection electron energy loss spectroscopy (REELS). The samples were surveyed first and then scanned for carbon and nitrogen specifically. Afterward, the measured spots (0.4 mm) were etched (for 30 s at 4000 eV with atom clusters of 1000 atoms) and then measured again to get results consisting of the bulk. The measurements were done using the flood gun to hinder charge build‐up in the sample. Nitrogen content (C/N) was calculated from individual elemental contents received by the XPS survey using Equation 3:

(3) $$C/N=\frac{x_{carbon}\;\left[at\;\%\right]}{x_{nitrogen}\;\left[at\;\%\right]}$$

The carbon electrodes were fabricated as free‐standing electrodes in a dry process. Therefore, the carbon powder was ground with 5 wt % polytetrafluoroethylene (PTFE) as a binder in an agate mortar at 80 °C until a dough‐like mass was obtained. It was rolled out to an electrode thickness between 150–200 μm. A disc cutter was used to achieve electrodes with a diameter of 10 mm, which were dried under vacuum at 80 °C for 24 h. The characterization took place in a symmetrical two‐electrode cell setup. The electrodes were pressed on titanium dioxide current collectors, which were coated with conductive carbon slurry (ElectroDAG EB012, HENKEL). As the separator, a WHATMANN GF/D filter with a 12 mm diameter was used.

The electrochemical measurements were performed with a BIOLOGIC VMP‐3 potentiostat/galvanostat using 1 m TEABF_4_/AcN as electrolyte. CV was conducted with varying scan rates from 5 to 500 mV s^−1^ and a potential range from 0 to 2.5 V. Potentiostatic electrochemical impedance spectroscopy (PEIS) was recorded with an amplitude of 10 mV in a frequency range from 10 mHz to 100 kHz. Galvanostatic charge–discharge with potential limitation (GCPL) was performed with a limit of 2.5 V and specific currents regarding the active carbon mass from 0.1 up to 15 A g^−1^. Between the charging and discharging an open‐circuit voltage (OCV) was applied for 10 s.

For CV, the specific capacitance (*C_spec_*) was calculated from the following Equation 4:

(4) $$C_{spec}=\frac{2\times I\left(t\right)}{\frac{dU}{dt}\times \frac{m_{act}}{2}}$$

where *I* is the strength of electric current, *U* the voltage, *t* the time and *m*_act_ denotes the active carbon mass without binder and conductive additive.

For galvanostatic charge and discharge curves, the specific capacitance was calculated from Equation 5:

(5) $$C_{spec}=\frac{2\times I}{\frac{dV}{dt}\times \frac{m_{act}}{2}}$$

## Conflict of interest

The authors declare no conflict of interest.

1

## Supporting information

As a service to our authors and readers, this journal provides supporting information supplied by the authors. Such materials are peer reviewed and may be re‐organized for online delivery, but are not copy‐edited or typeset. Technical support issues arising from supporting information (other than missing files) should be addressed to the authors.

Supporting Information

## Data Availability

The data that support the findings of this study are available from the corresponding author upon reasonable request.
