# Supplementary material for: Scale‐Up of Solvent‐Free, Mechanochemical Precursor Synthesis for Nanoporous Carbon Materials via Extrusion
Source: ChemSusChem. 2022 Jun 28;15(16):e202200651. doi: 10.1002/cssc.202200651 (PMC9543152; doi:10.1002/cssc.202200651)
Supplement: Supplementary file 1 — Supporting Information [file CSSC-15-0-s001.pdf]

ChemSusChem

Supporting Information

Scale-Up of Solvent-Free, Mechanochemical Precursor Synthesis for Nanoporous Carbon Materials via Extrusion

Tilo Rensch, Viviene Chantrain, Miriam Sander, Sven Grätz, and Lars Borchardt*© 2022 The Authors. ChemSusChem published by Wiley-VCH GmbH. This is an open access article under the terms of the Creative Commons Attribution License, which permits use, distribution and reproduction in any medium, provided the original work is properly cited.

Table of Contents

1. Optimization of Extrusion parameters iii

2 Characterization iv

Scanning Electron Microscopy iv

Electron dispersive X-ray spectroscopy v

Infrared spectroscopy viii

X-Ray photoelectron spectroscopy xi

Argon physisorption xxiii

Differential scanning calorimetry xxiv

X-ray diffraction xxv

Nitrogen physisorption xxviii

Space-time yield xxxv

Electrochemical analysis xxxvi

1. OPTIMIZATION OF EXTRUSION PARAMETERS

Table S1. Optimization of extrusion parameters with respect to Carbon yield, Nitrogen content and specific surface calculated from N_2 physisorption (SSA).

Entry	Sample ID	Screw speed [rpm]	Temperature [°C]	Feed rate [g min^{-1}]	C-yield [%]	C/N	SSA [m^2 g^{-1}]
1	LUK-1	55	25	1	3.9	41.0	3211
2	LUK-2	55	50	1	6.9	91.6	3380
3	LUK-3	55	80	1	10.4	15.0	2520
4	LUK-4	55	100	1	13.1	32.5	3326
5	LUK-5	55	120	1	15.5	72.3	2762
6	LUK-6	45	100	1	11.0	53.8	2804
7	LUK-7	75	100	1	11.5	37.7	2943
8	LUK-8	95	100	1	14.8	35.5	2773
9	LUK-9	150	100	1	10.5	76.3	3037
10	LUK-10	55	100	1.5	17.2	70.9	3126
11	LUK-11	55	100	2	12.5	37.8	3857
12	LUK-12	55	100	5	2.0	17.3	2739

Table S2. Alteration of Nitrogen sources at the set screw speed of 55 rpm and a screw speed of 1 g/min. LUK=Lignin-Urea-K_2CO_3, LMK=Lignin-Melmine-K_2CO_3, LBK=Lignin-Biuret-K_2CO_3. Temperature was increased until the lowest decomposition temperature of the mixture was reached.

Entry	Sample ID	Temperature [°C]	C-yield [%]	C/N	SSA [m^2 g^{-1}]
1	LMK-1	100	3.7	17.0	2934
2	LMK-2	140	4.2	19.9	3165
3	LMK-3	300	6.4	17.3	3316
4	LBK-1	100	6.2	56.5	3384
5	LBK-2	180	14.1	142.3	2962

Table S3. Comparison between extruded LUK mixture, ball milled LUK mixture, LUK made with a screw only containing conveying elements, reaction mixture without urea and wood waste as feedstock.

Entry	Sample ID	Unique feature deviating from entry 1	C-yield [%]	C/N	SSA [m^2 g^{-1}]
1	LUK-4	-	13.1	32.5	3326
2	LUK-BM	Ball mill	5.2	15.1	2575
3	LUK-CS	Conveying screws	12.2	69.4	2393
4	LK	Without Urea	21.2	140.9	584
5	WUK	Alternative feedstock	8.5	29.0	2581

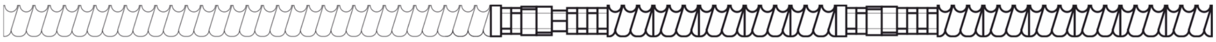

Figure S1. Screw setup for extrusion consisting of three conveying zones and two kneading zones with varying kneading blocks.

2 CHARACTERIZATION

Scanning Electron Microscopy

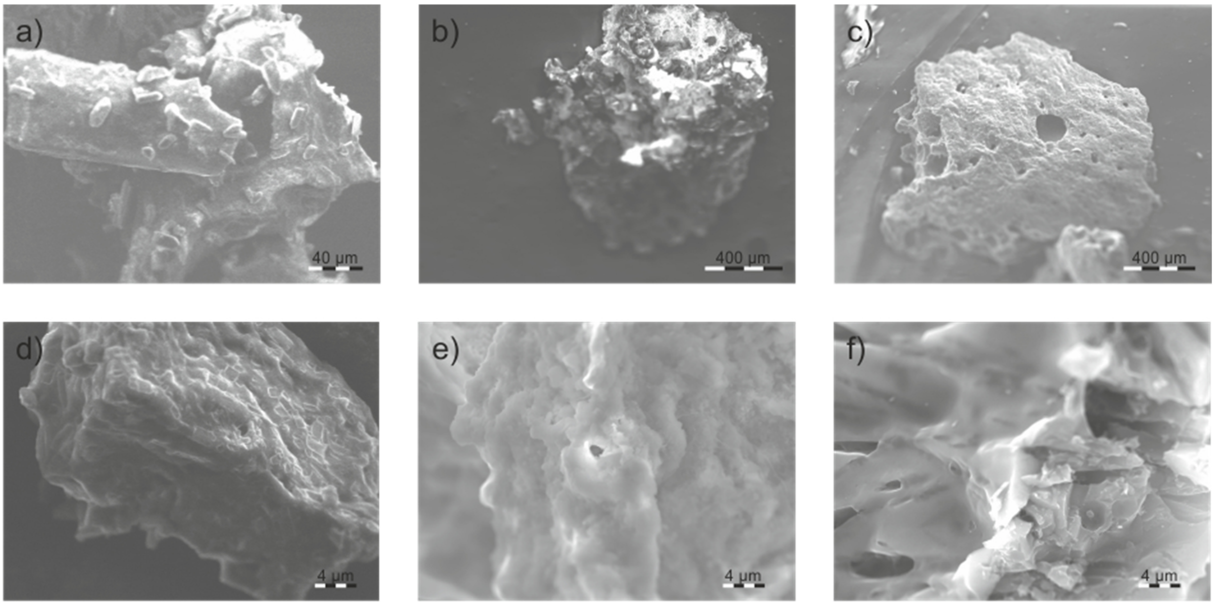

Figure S2. Images taken with scanning electron microscopy. a) LUK-4 polymer after extrusion, 450 times magnification. b) LUK-4 carbon after pyrolysis, 65 times magnification. c) LUK-4 carbon after pyrolysis and washing, 60 times magnification. d) LUK-4 polymer after extrusion, 3500 times magnification. e) LUK-4 carbon after pyrolysis, 3500 times magnification. f) LUK-4 carbon after pyrolysis and washing, 3500 times magnification.

Electron dispersive X-ray spectroscopy

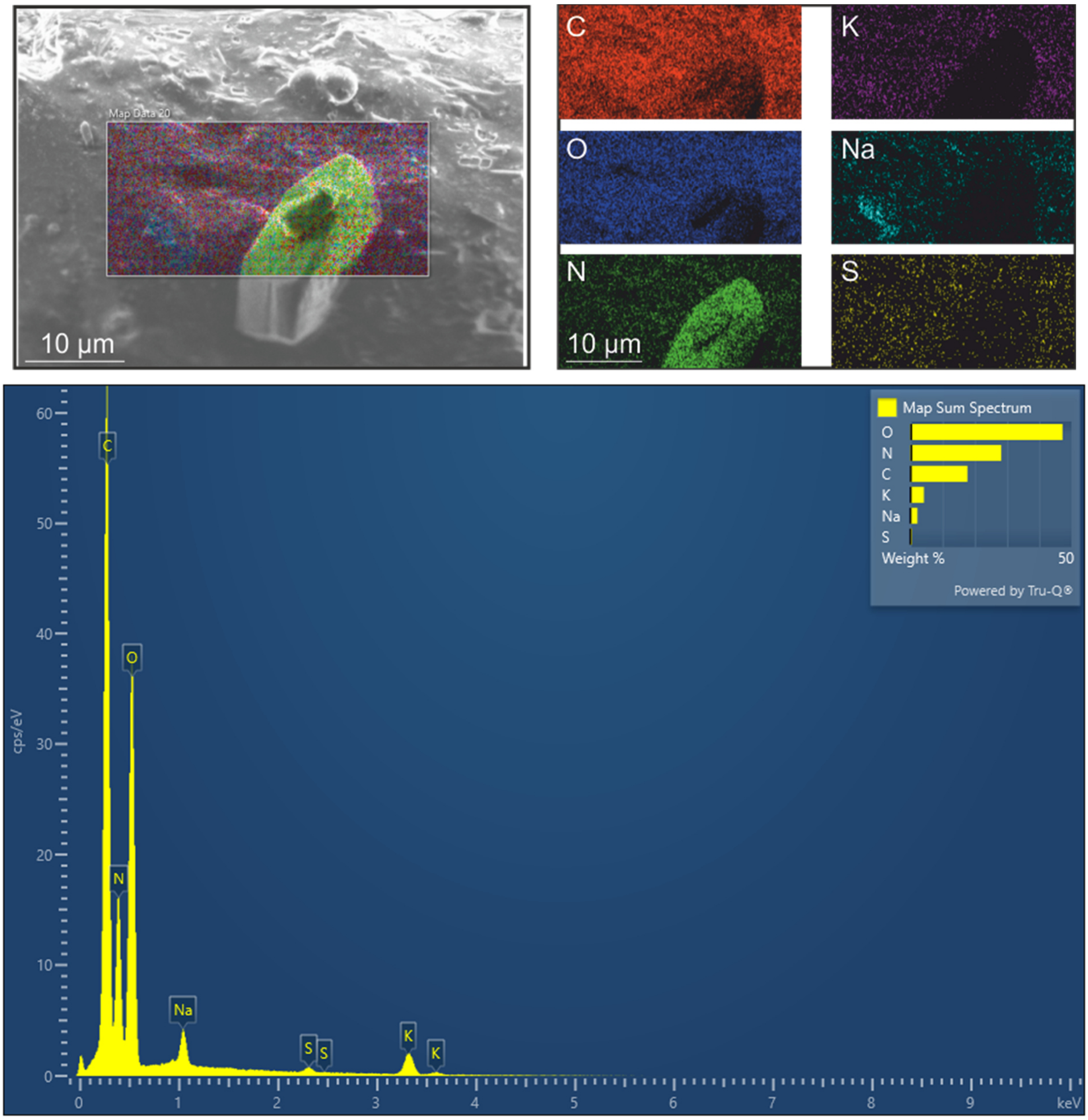

Figure S3. Electron dispersive X-ray spectroscopy mapping of LUK-4 polymer after extrusion. A combined image is displayed in the top left, element specific maps in the top right. The bottom image shows the full spectrum.

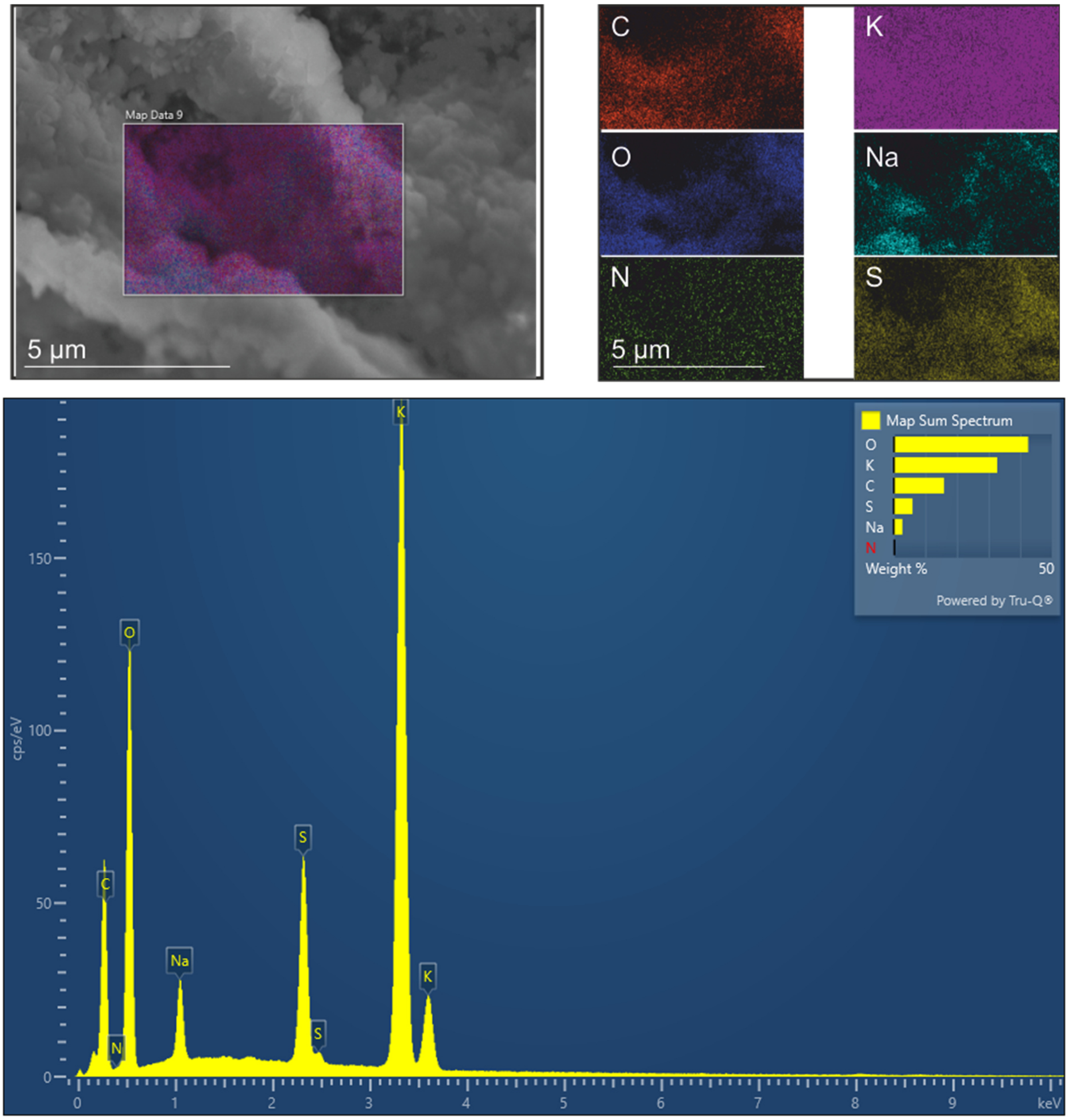

Figure S4. Electron dispersive X-ray spectroscopy mapping of LUK-4 carbon after pyrolysis. A combined image is displayed in the top left, element specific maps in the top right. The bottom image shows the full spectrum.

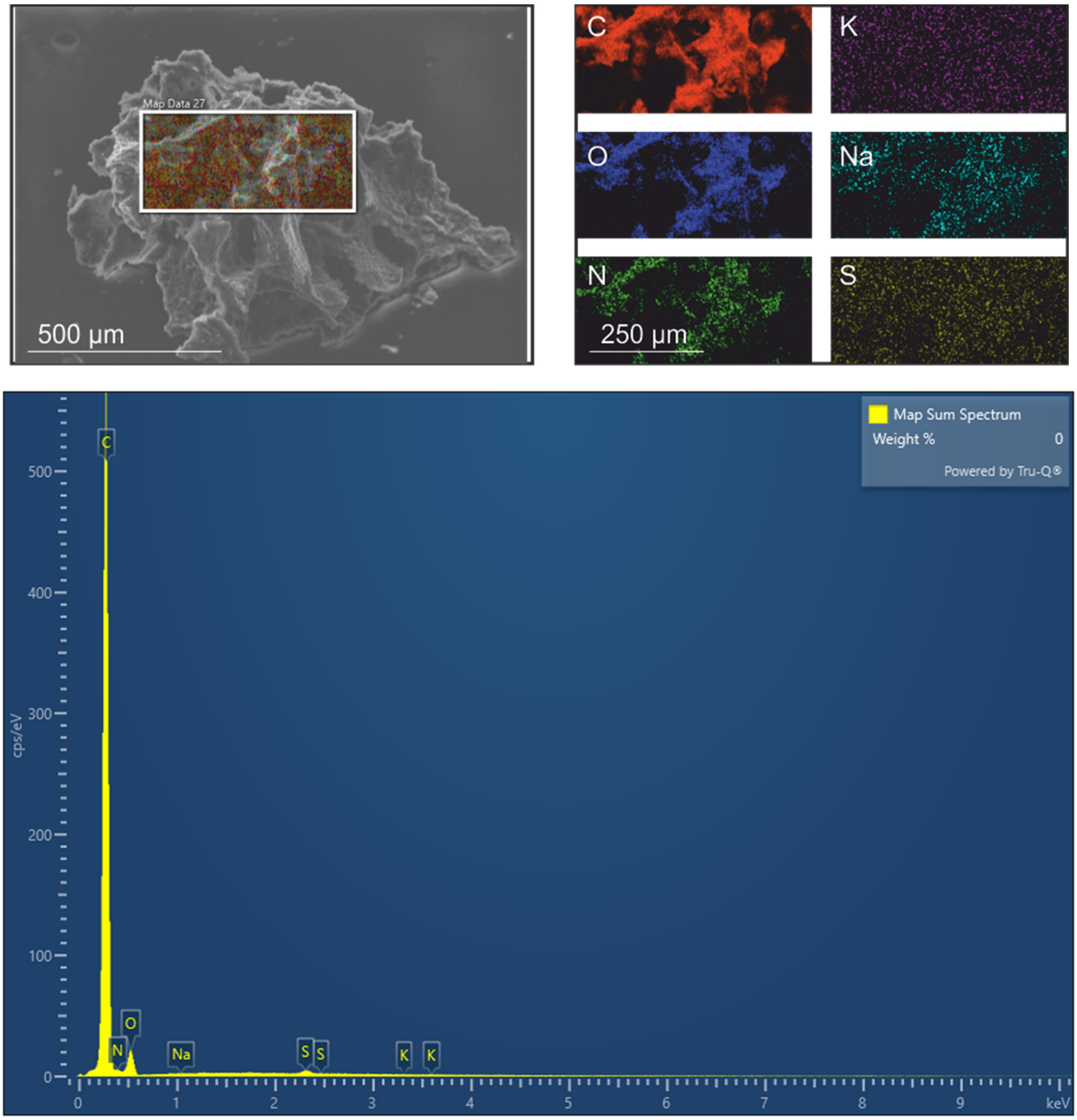

Figure S5. Electron dispersive X-ray spectroscopy mapping of LUK-4 carbon after pyrolysis and washing with hydrochloric acid. A combined image is displayed in the top left, element specific maps in the top right. The bottom image shows the full spectrum.

Infrared spectroscopy

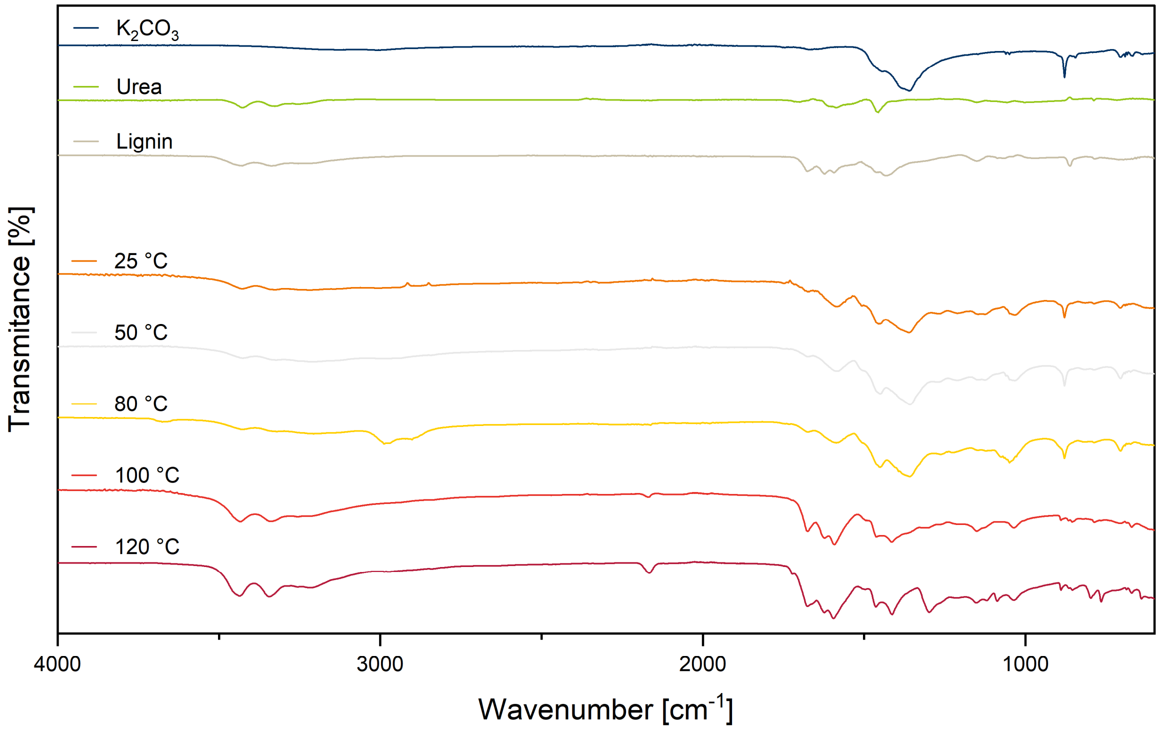

Figure S6. FTIR-Spectra of temperature varied polymer samples prior to pyrolysis.

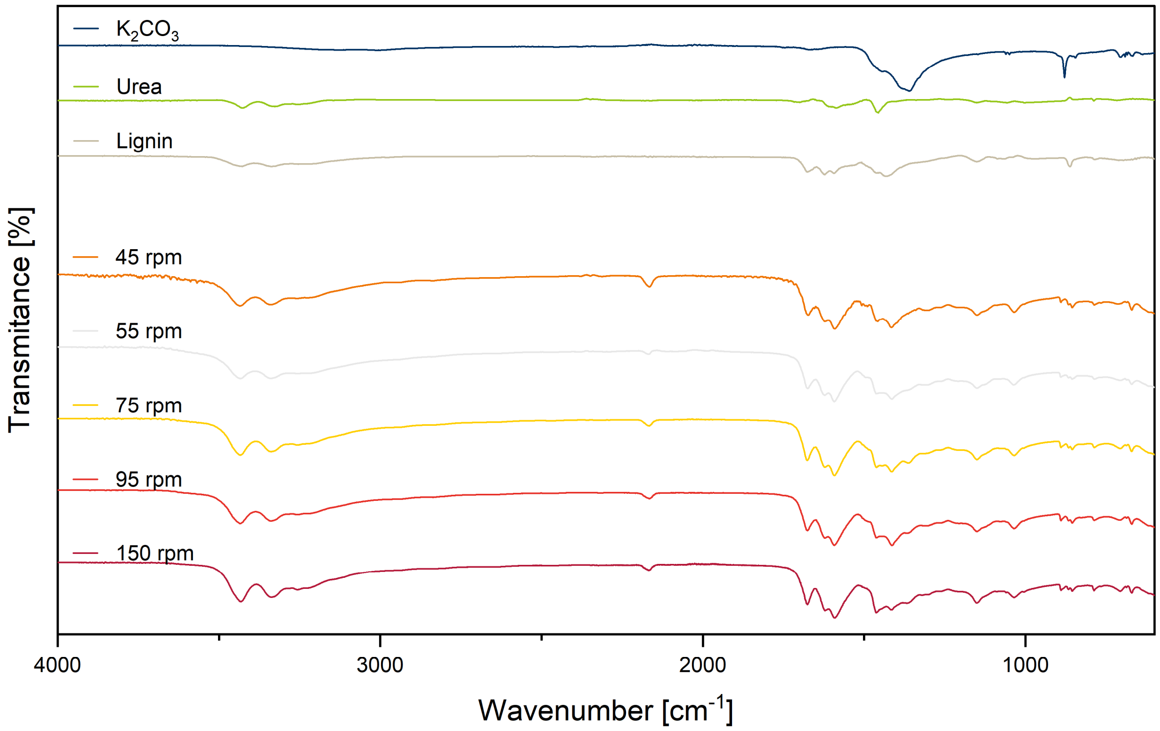

Figure S7. FTIR-Spectra of rotational speed varied polymer samples prior to pyrolysis.

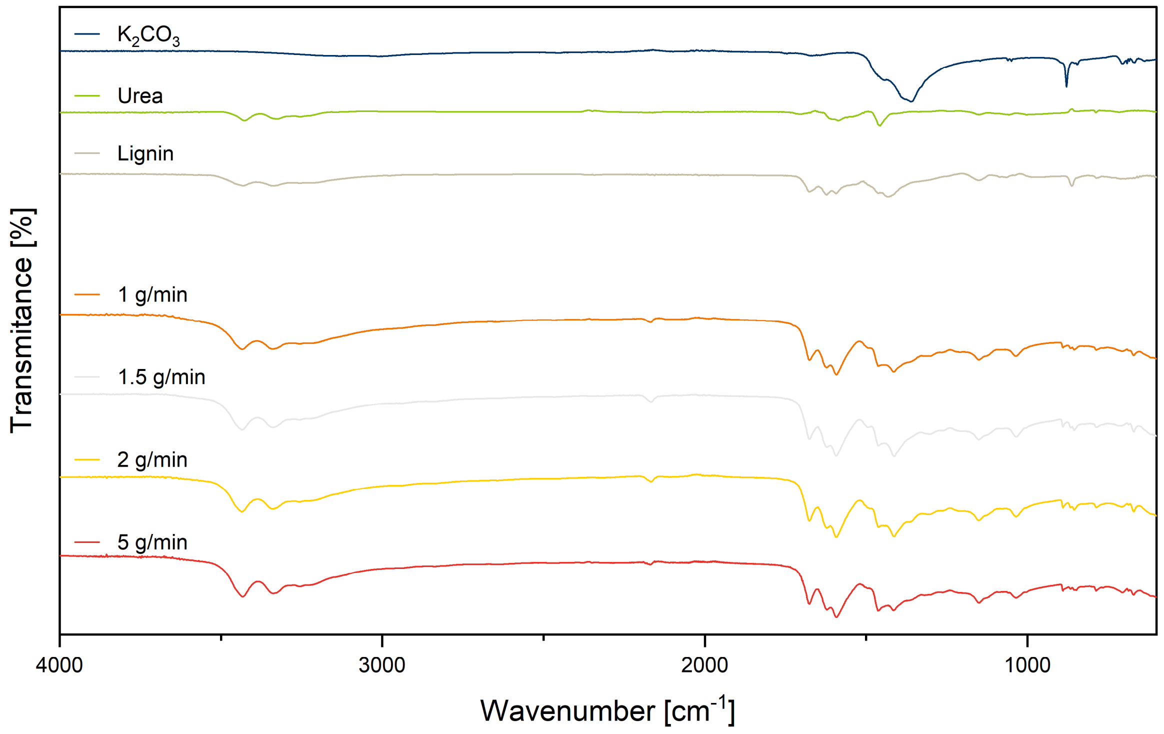

Figure S8. FTIR-Spectra of feed rate varied polymer samples prior to pyrolysis.

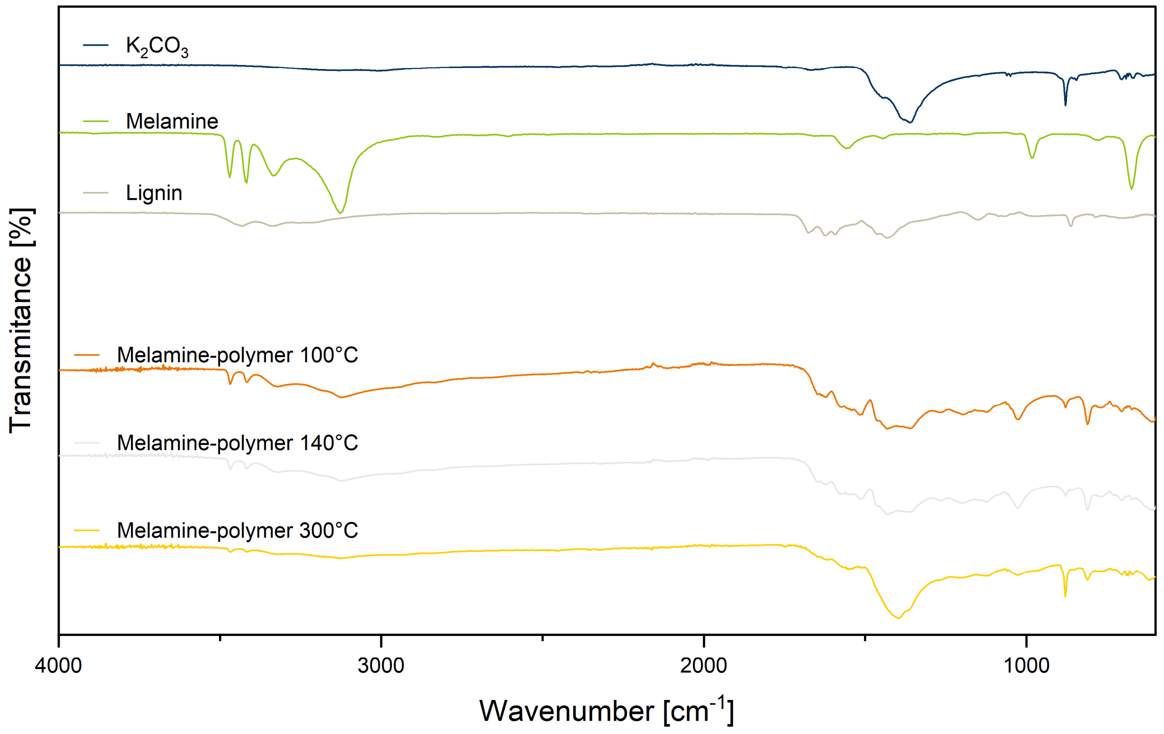

Figure S9. FTIR-Spectra of melamine-based polymer samples prior to pyrolysis.

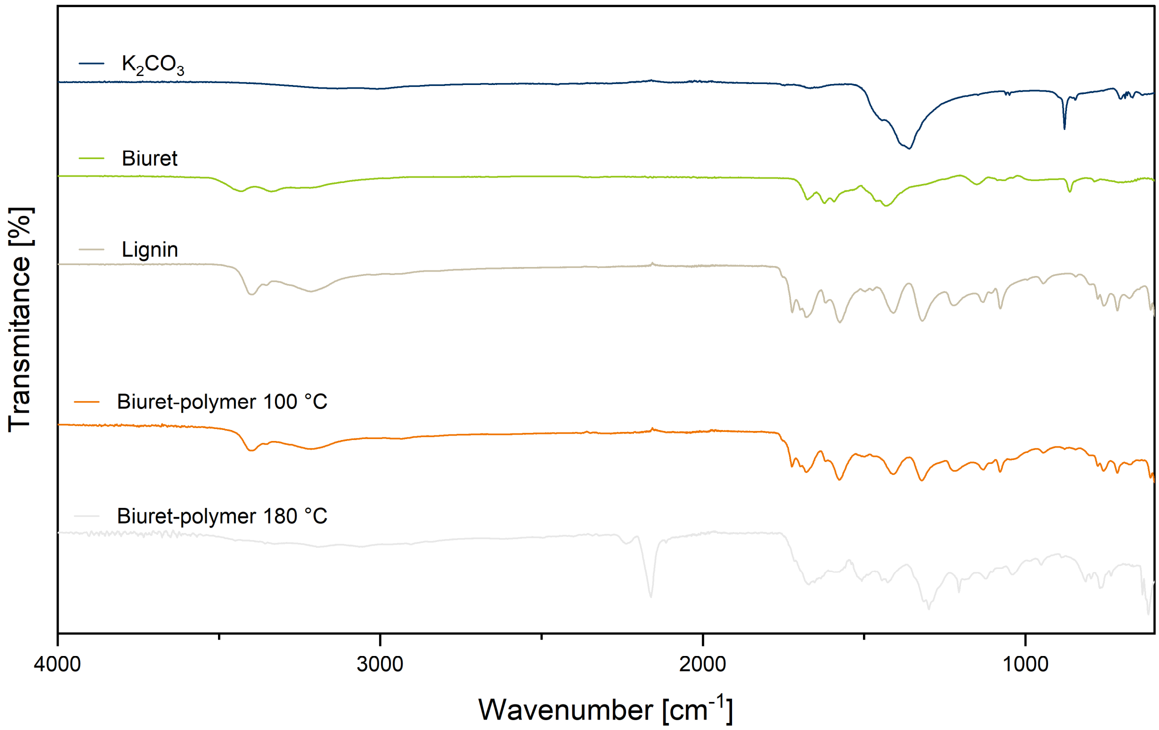

Figure S10. FTIR-Spectra of biuret-based polymer samples prior to pyrolysis.

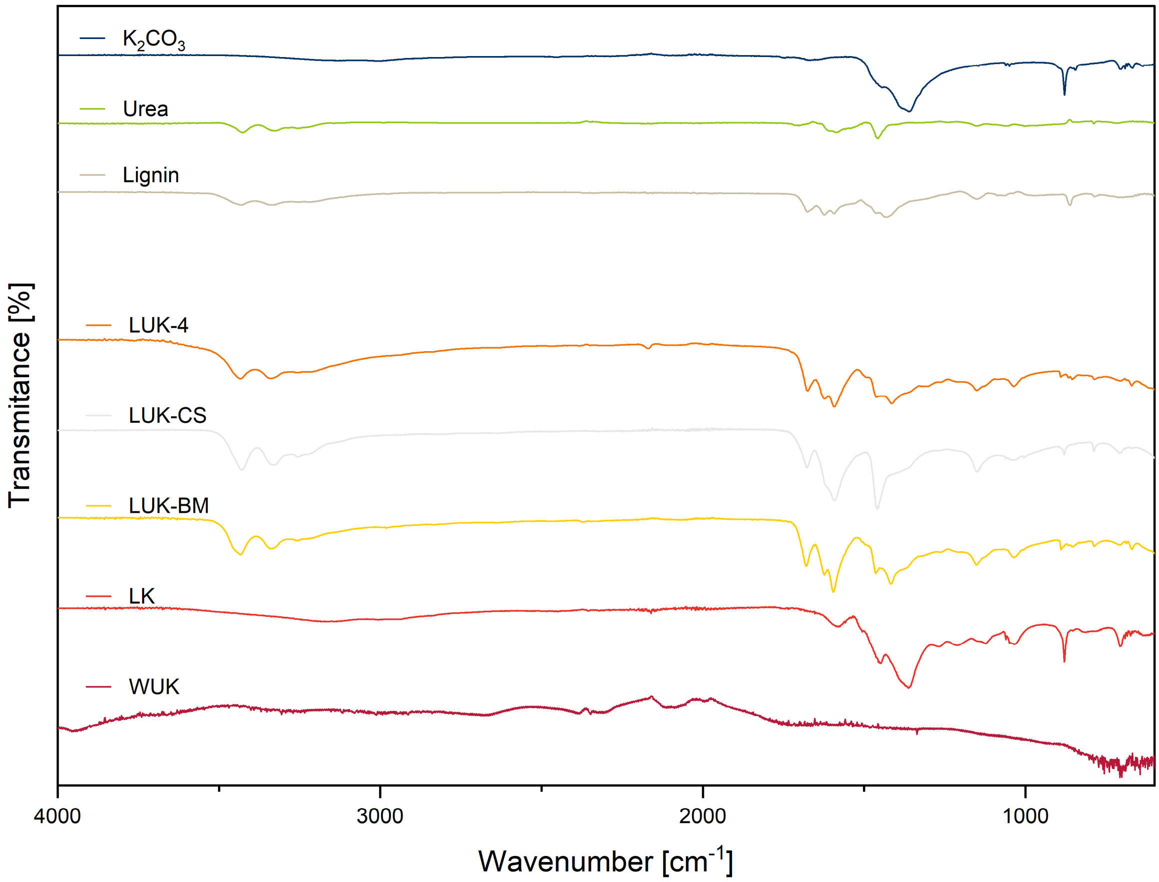

Figure S11. FTIR-Spectra of reference polymer samples prior to pyrolysis.

X-Ray photoelectron spectroscopy

Table S4. Elemental composition of samples as determined by XPS. Potassium, Sodium and Sulfur were only detected for crude materials (before washing).

Entry	Sample ID	C-content [at%]	N-content [at%]	O-content [at%]	K-content [at%]	Na-content [at%]	S-content [at%]
1	LUK-1	93.86	2.29	3.85	-	-	-
2	LUK-2	95.29	1.04	3.67	-	-	-
3	LUK-3	84.59	5.64	9.77	-	-	-
4	LUK-4	89.72	2.76	7.52	-	-	-
5	LUK-5	91.12	1.26	7.62	-	-	-
6	LUK-6	88.17	1.64	10.19	-	-	-
7	LUK-7	88.26	2.34	9.4	-	-	-
8	LUK-8	89.36	2.52	8.12	-	-	-
9	LUK-9	87.79	1.15	11.05	-	-	-
10	LUK-10	86.48	1.22	12.3	-	-	-
11	LUK-11	88.51	2.34	9.15	-	-	-
12	LUK-12	88.24	5.11	6.65	-	-	-
13	LUK-4 polymer	51.08	13.76	26.01	7.63	0.75	0.76
14	LUK-4 crude	23.97	0.99	46.91	20.92	5.80	1.41
15	LMK-1	88.87	5.23	5.9	-	-	-
16	LMK-2	89.84	4.52	5.64	-	-	-
17	LMK-3	90.81	5.25	3.95	-	-	-
18	LBK-1	93.77	1.66	4.56	-	-	-
19	LBK-2	93.89	0.66	5.45	-	-	-
20	LUK-BM	87.51	5.78	6.71	-	-	-
21	LUK-CS	89.53	1.29	9.19	-	-	-
22	LK	92.98	0.66	6.36	-	-	-
23	WUK	88.93	3.07	7.98	-	-	-

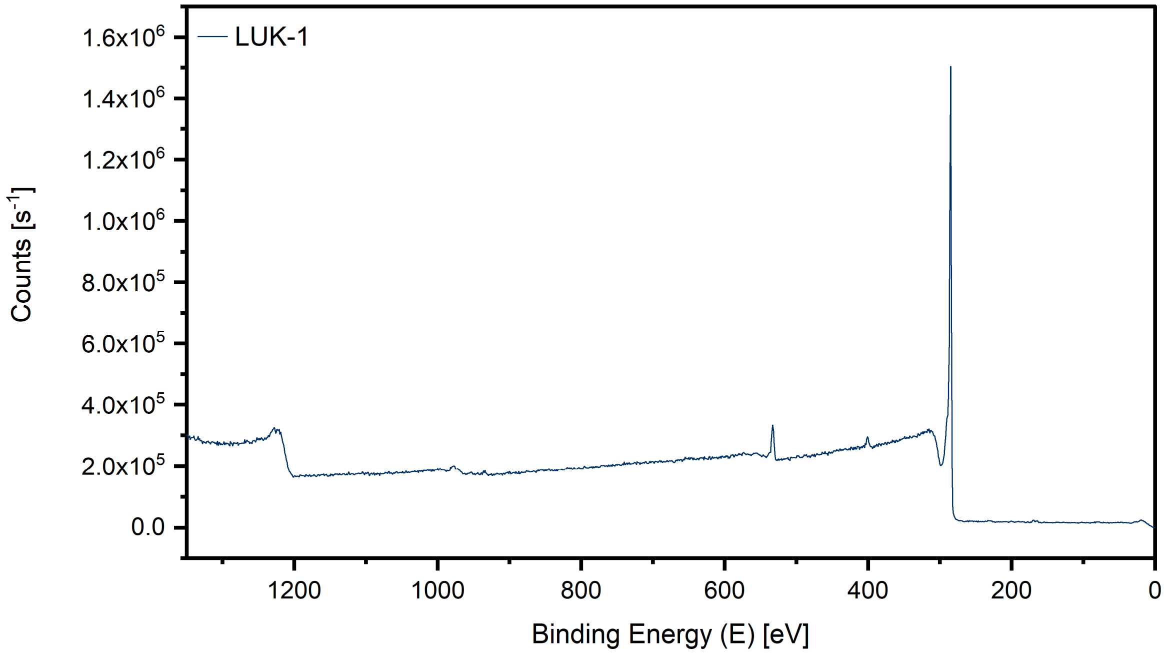

Figure S12. XPS survey of sample LUK-1.

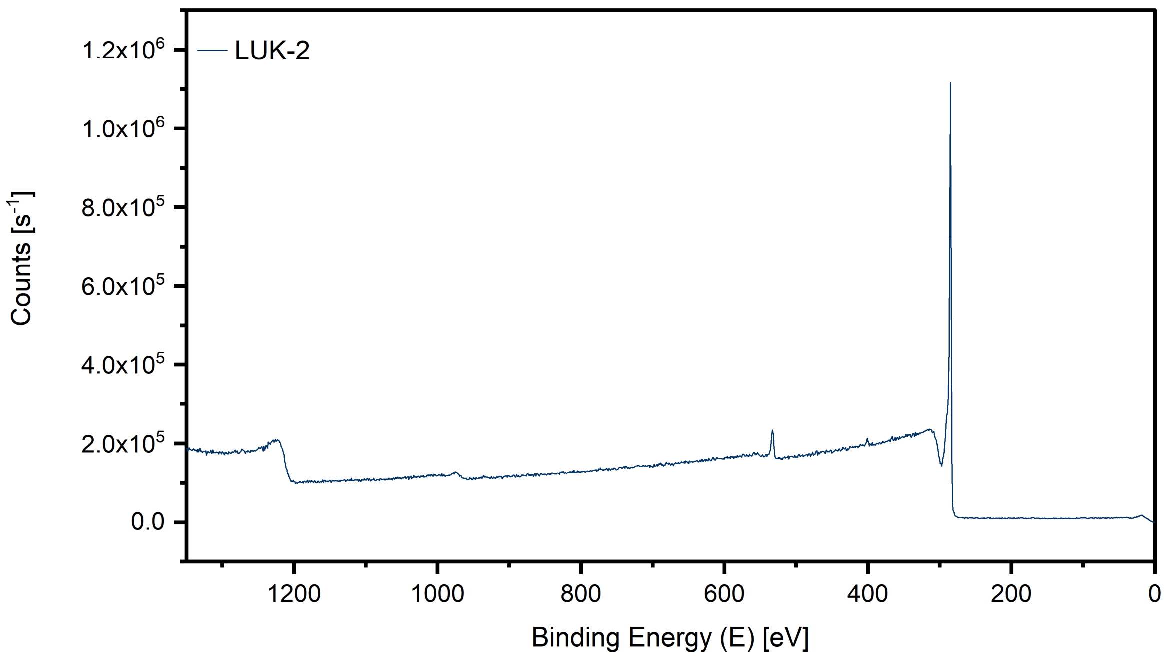

Figure S13. XPS survey of sample LUK-2.

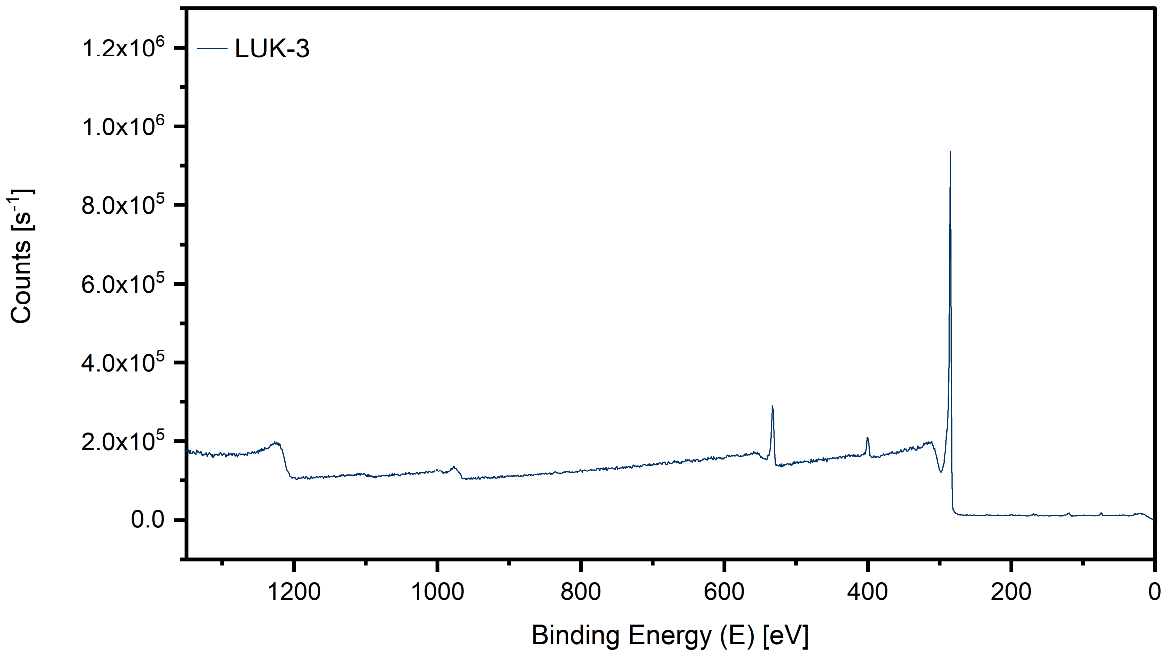

Figure S14. XPS survey of sample LUK-3.

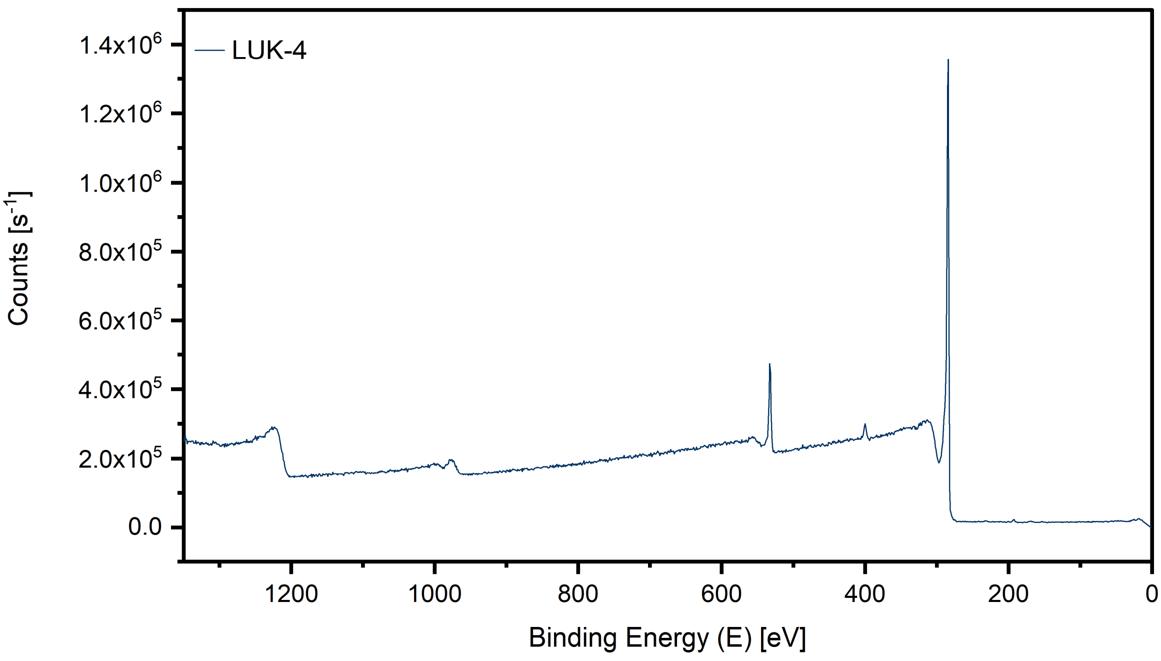

Figure S15. XPS survey of sample LUK-4.

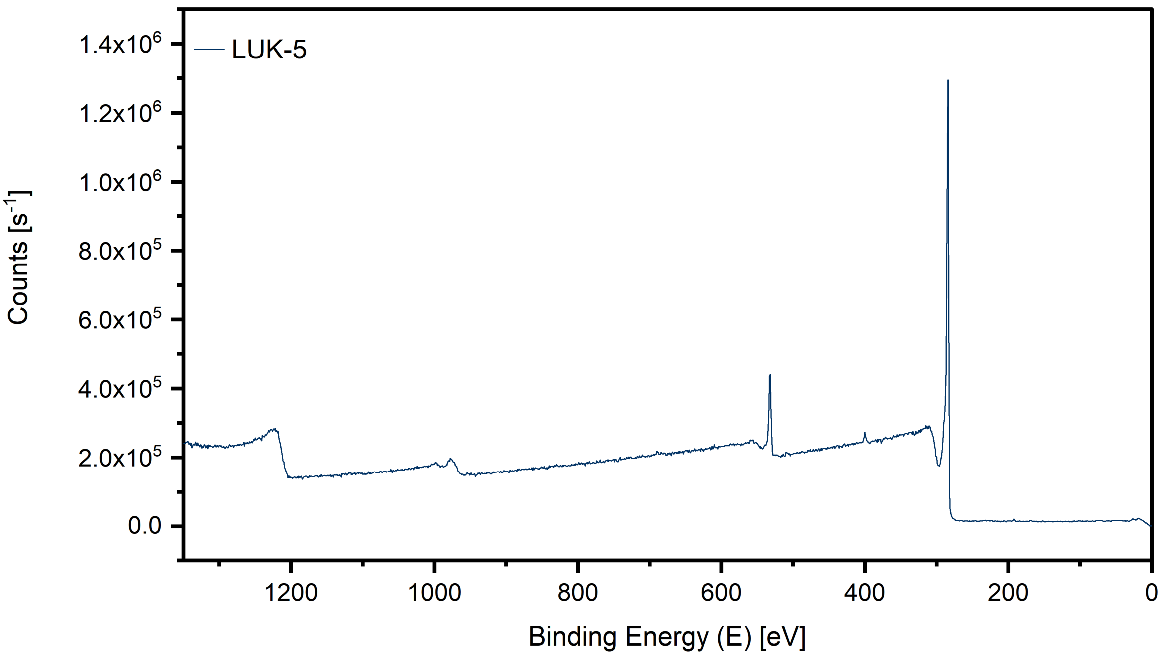

Figure S16. XPS survey of sample LUK-5.

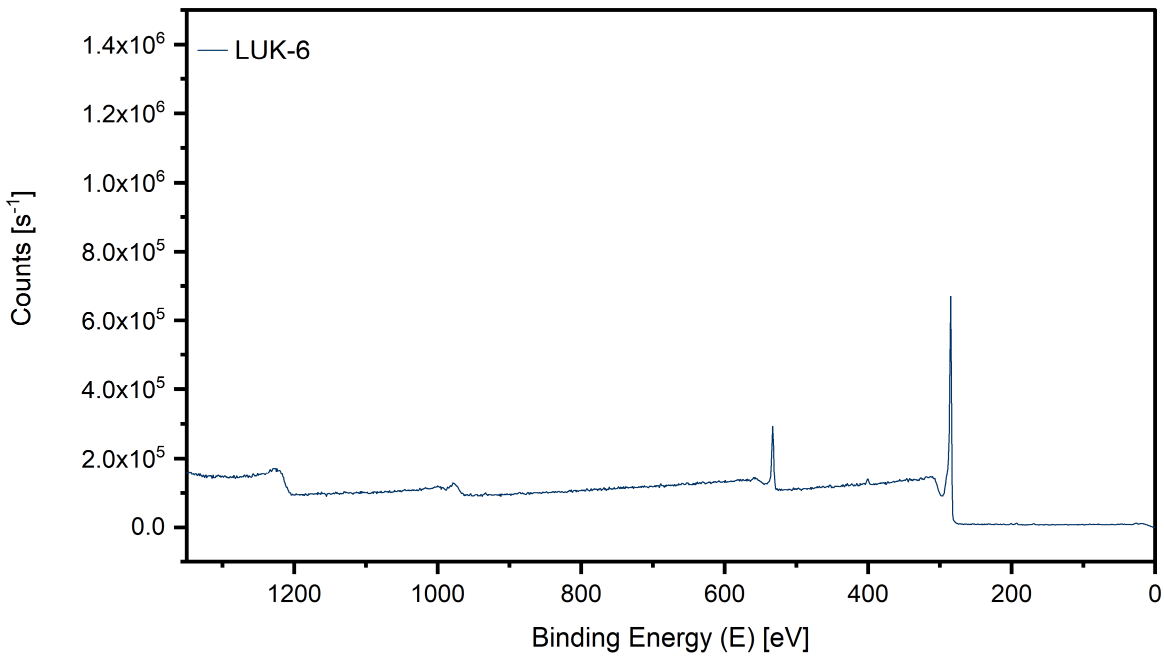

Figure S17. XPS survey of sample LUK-6.

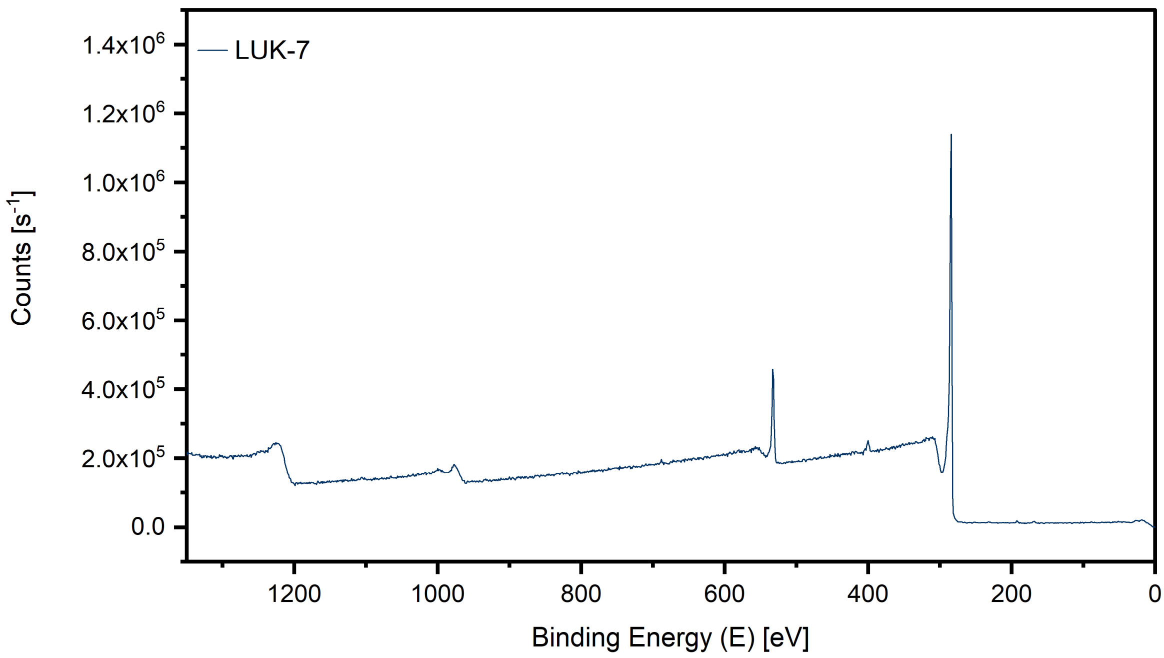

Figure S18. XPS survey of sample LUK-7.

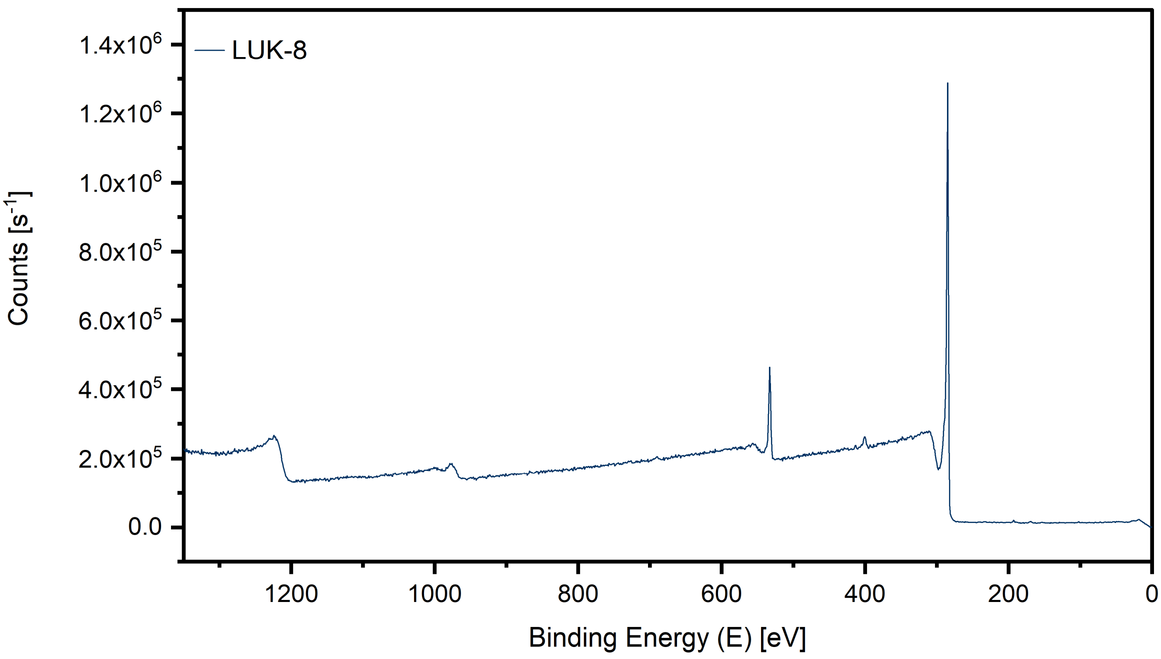

Figure S19. XPS survey of sample LUK-8.

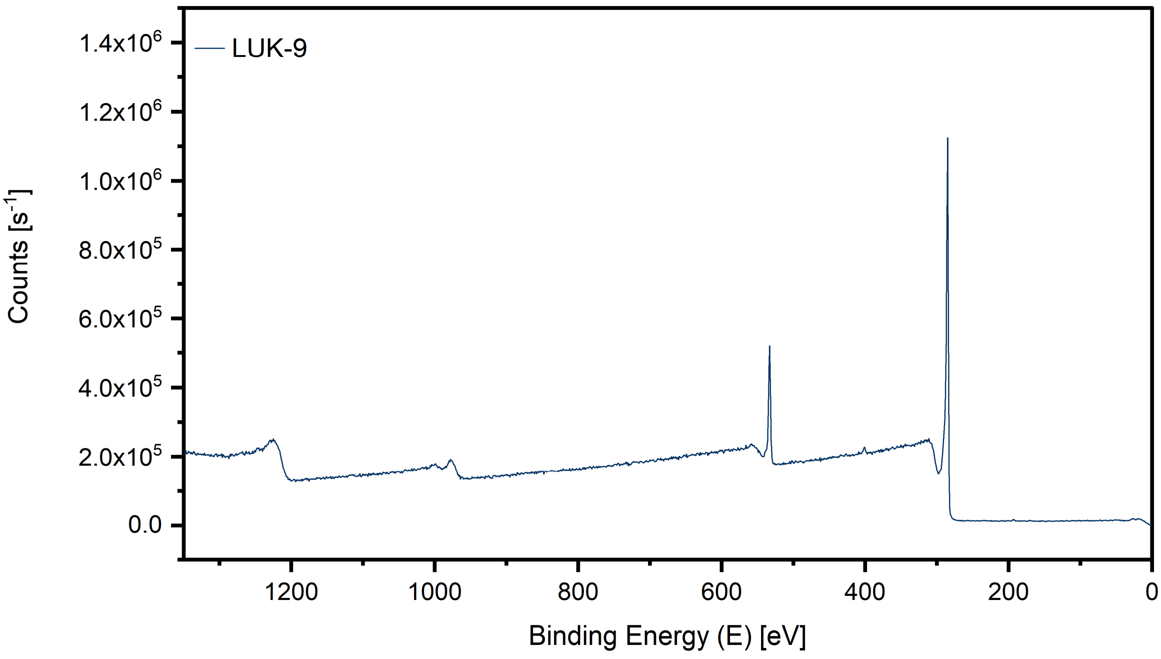

Figure S20. XPS survey of sample LUK-9.

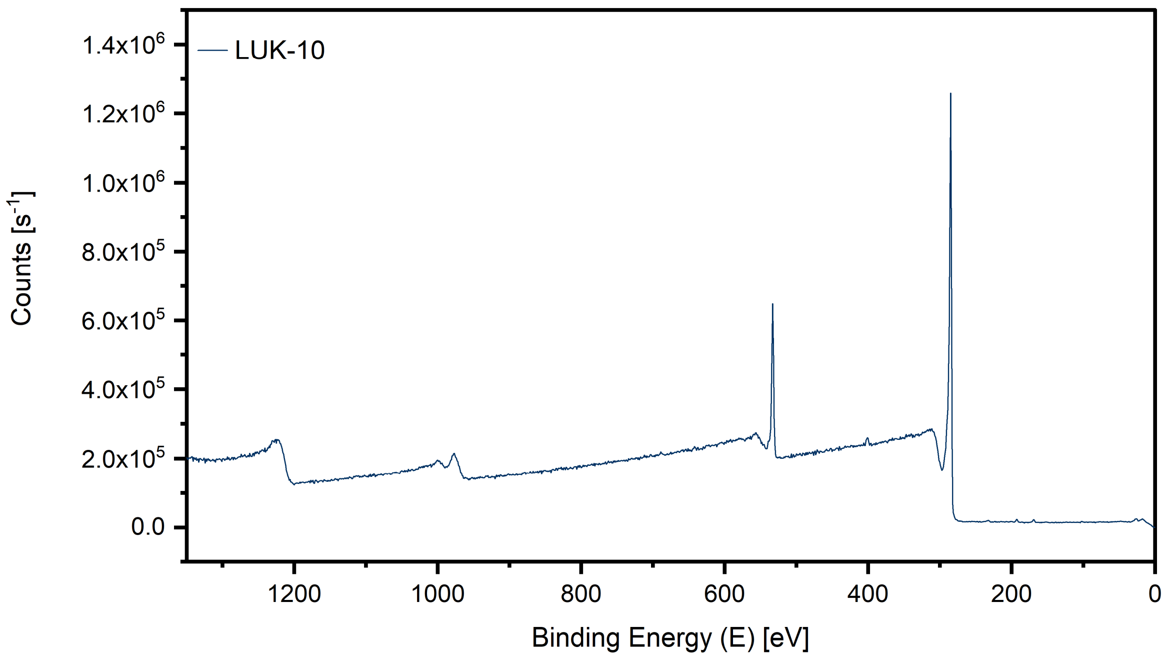

Figure S21. XPS survey of sample LUK-10.

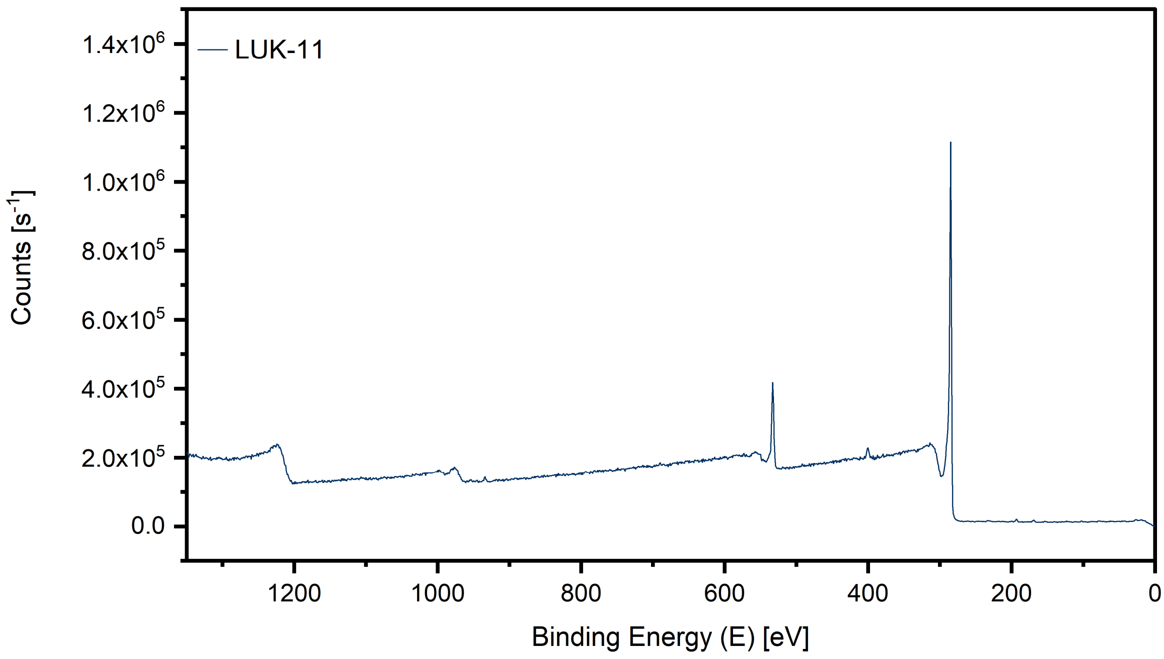

Figure S22. XPS survey of sample LUK-11.

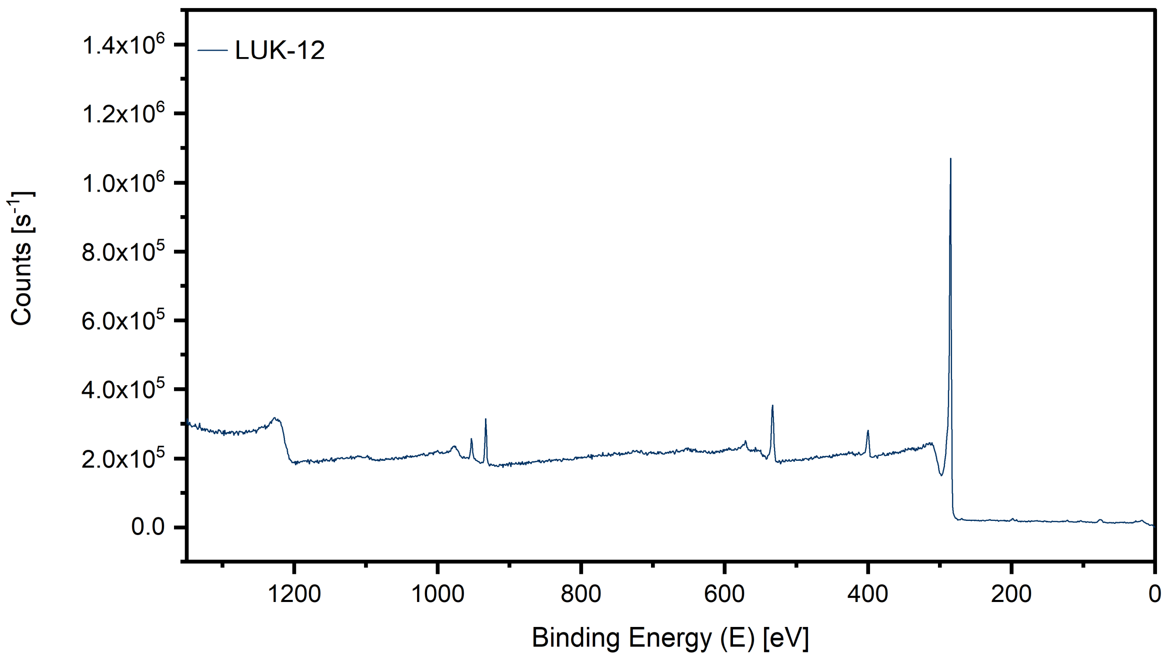

Figure S23. XPS survey of sample LUK-12.

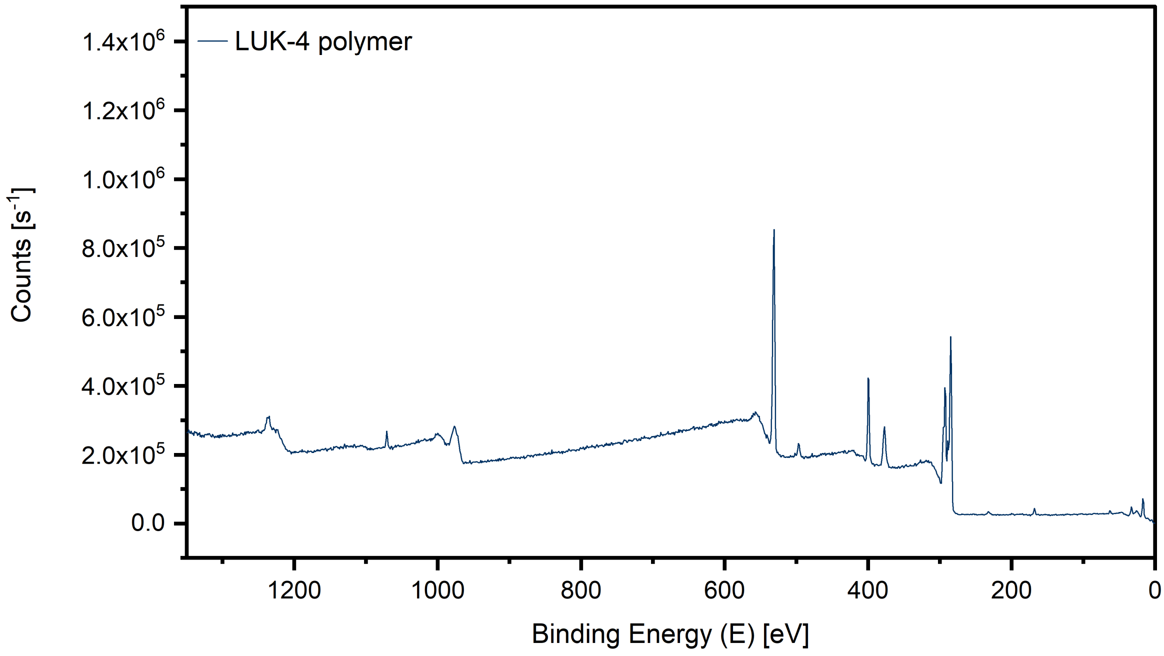

Figure S24. XPS survey of sample LUK-4 polymer.

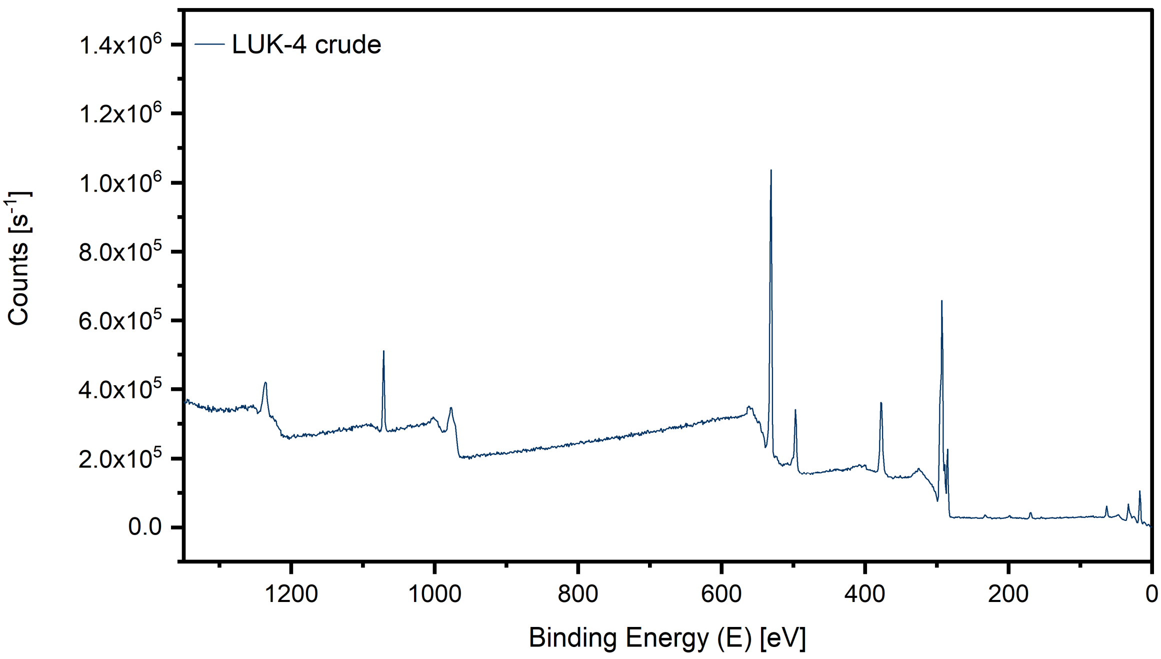

Figure S25. XPS survey of sample LUK-4 crude.

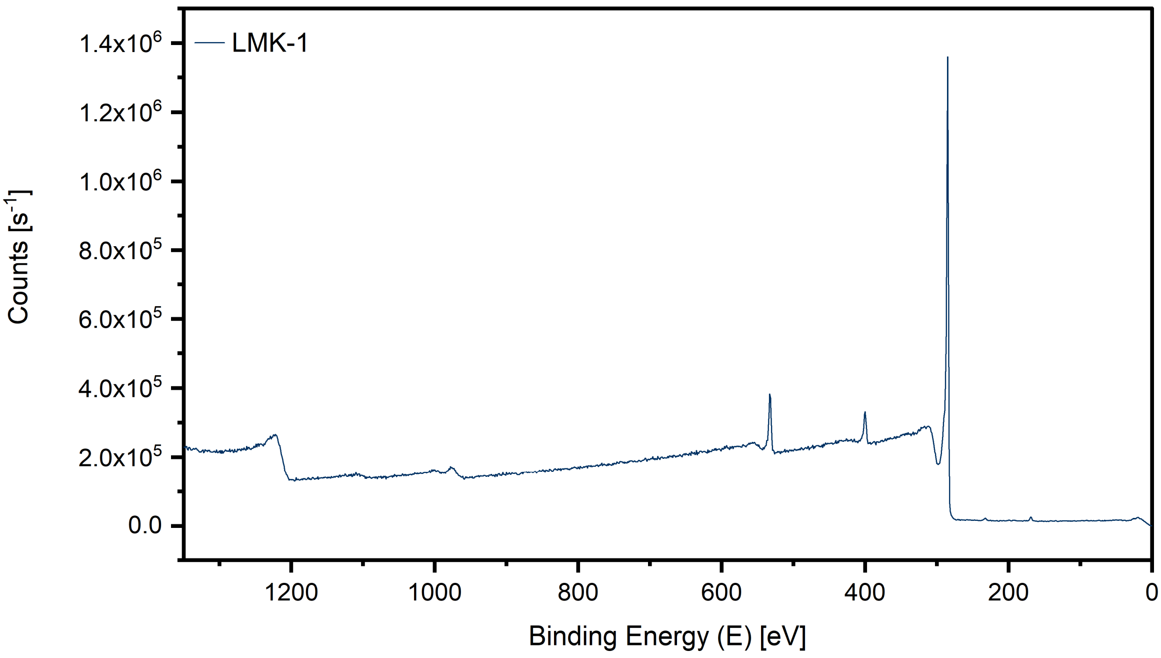

Figure S26. XPS survey of sample LMK-1.

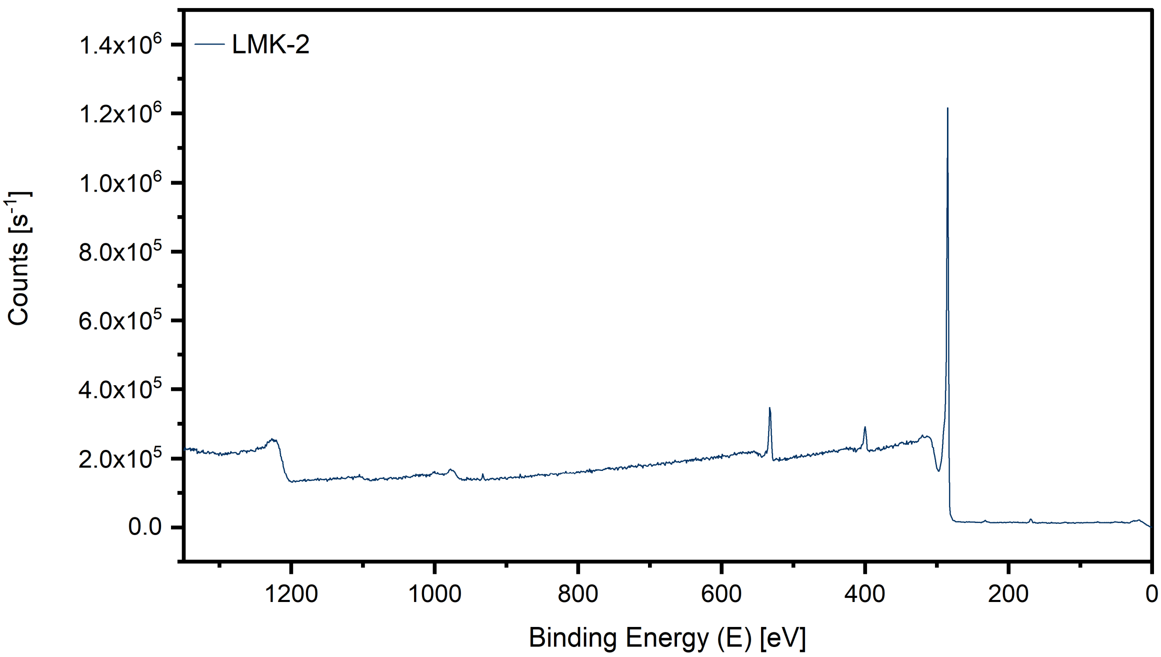

Figure S27. XPS survey of sample LMK-2.

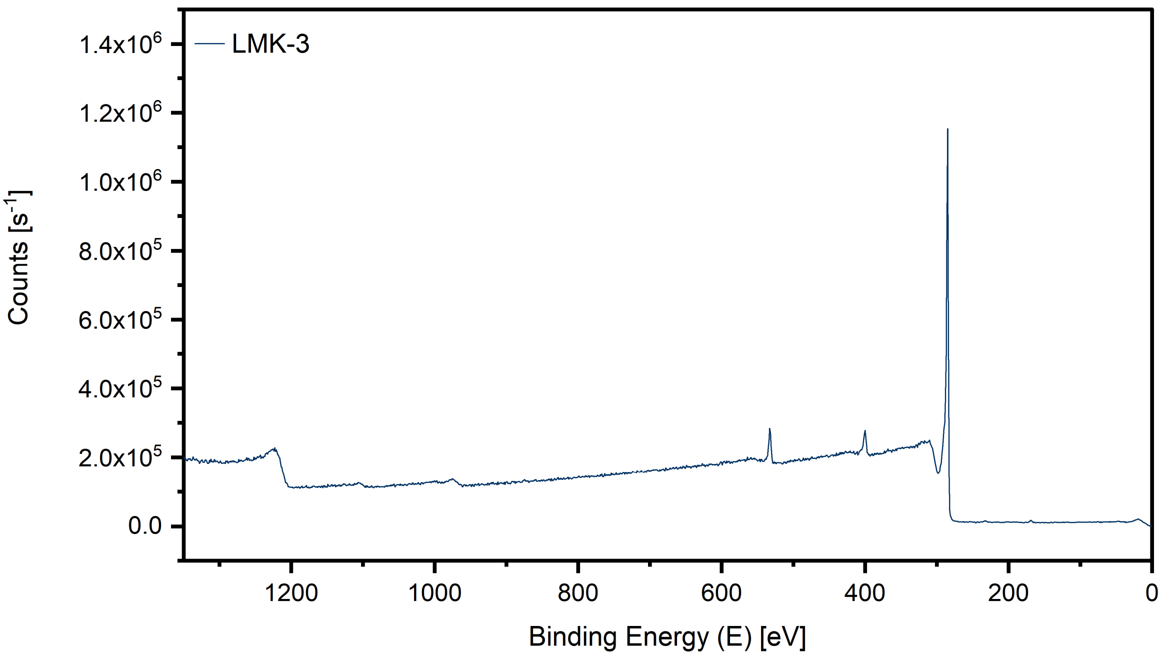

Figure S28. XPS survey of sample LMK-3.

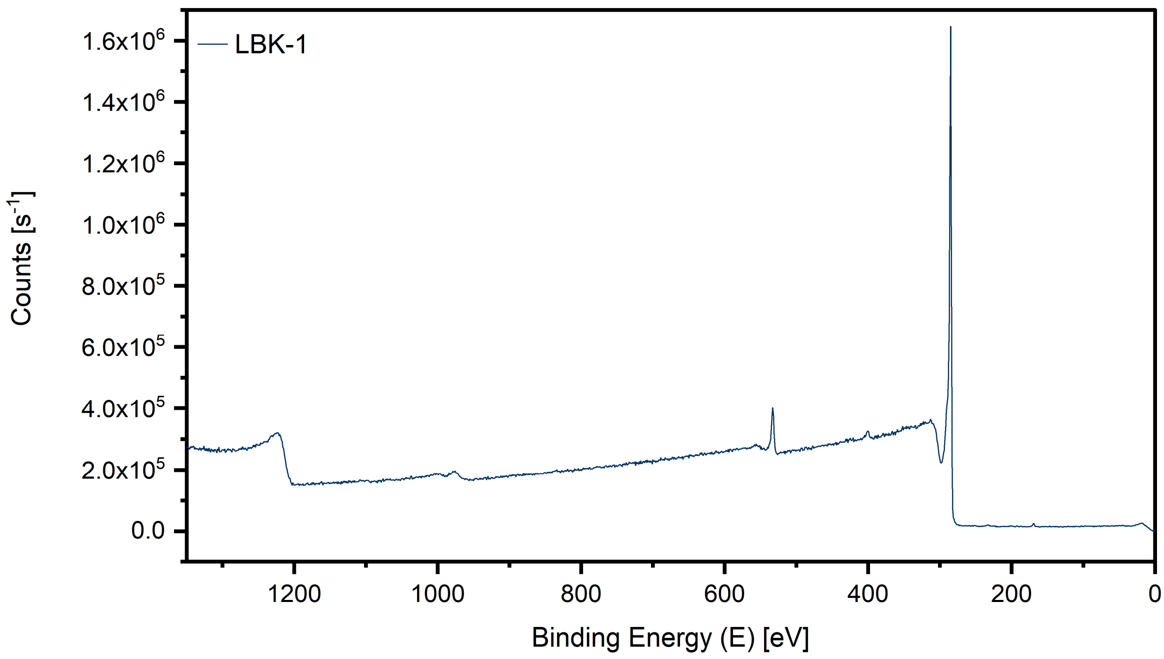

Figure S29. XPS survey of sample LBK-1.

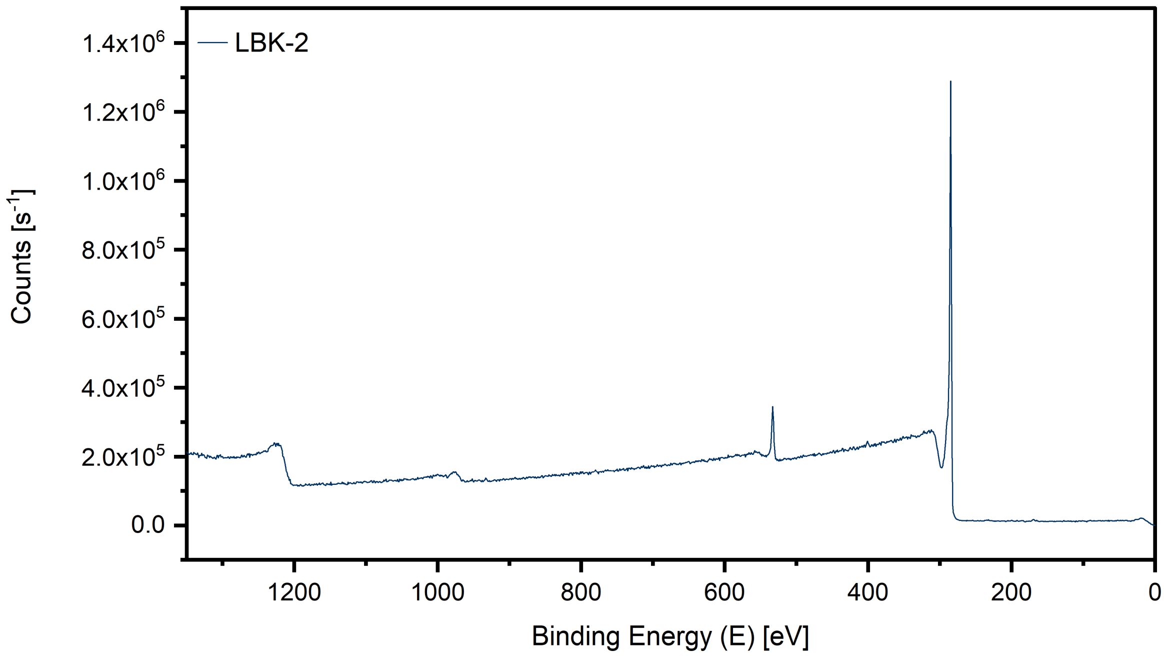

Figure S30. XPS survey of sample LBK-2.

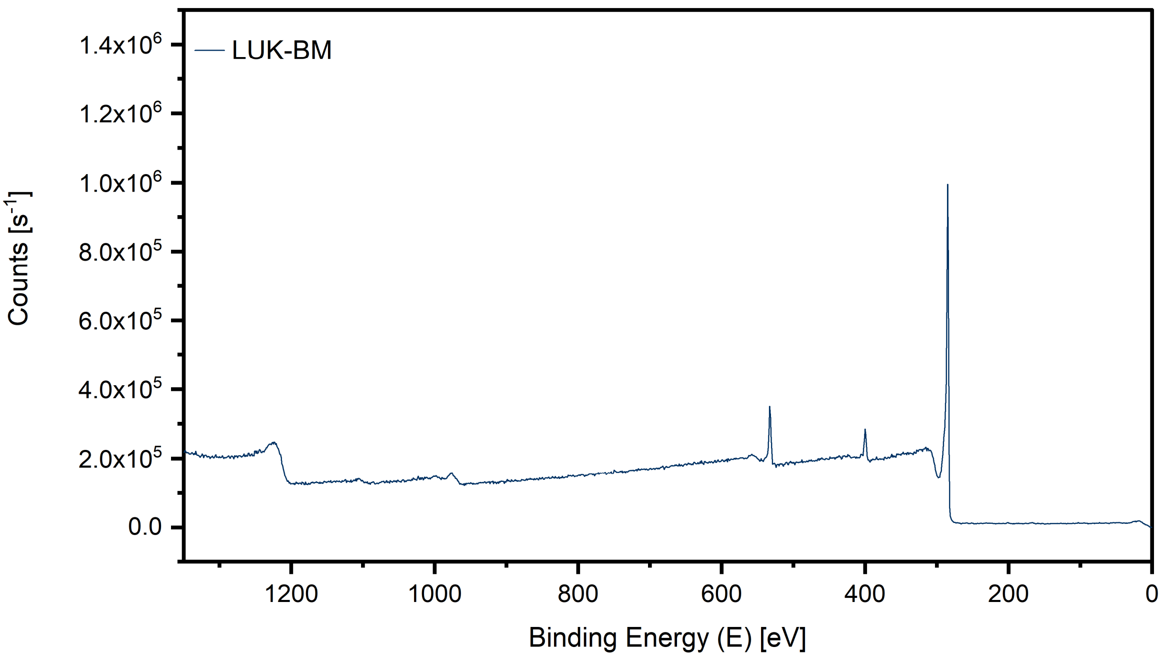

Figure S31. XPS survey of sample LUK-BM.

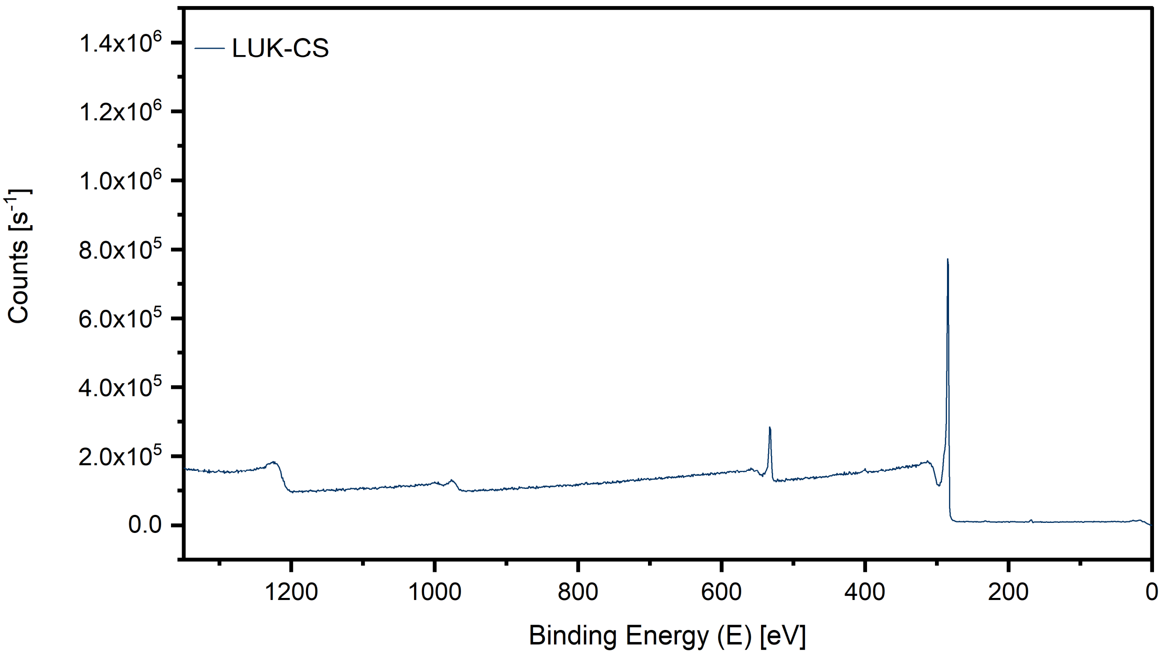

Figure S32. XPS survey of sample LUK-CS.

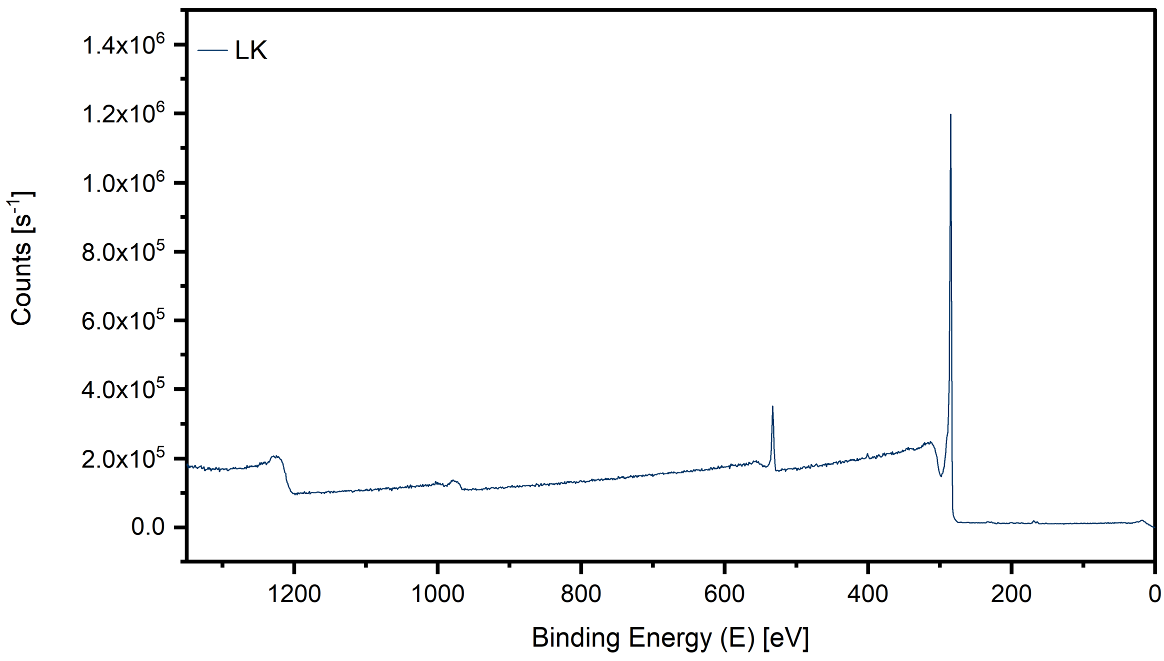

Figure S33. XPS survey of sample LK.

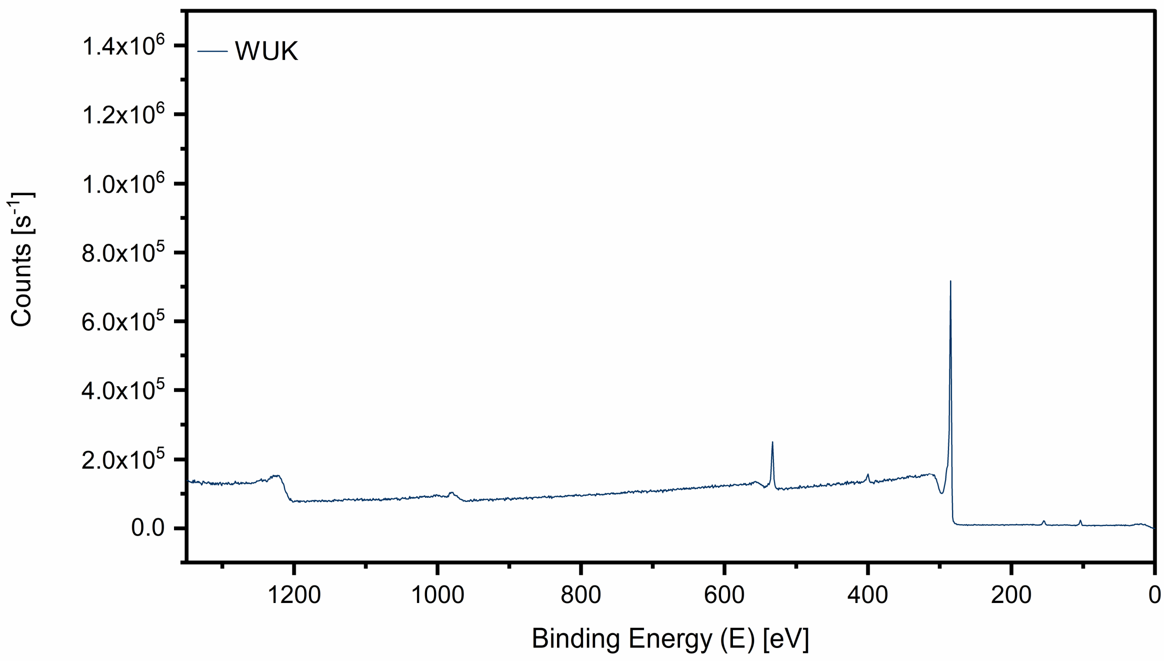

Figure S34. XPS survey of sample WUK.

Argon physisorption

Table S5: Results of Ar-physisorption analysis of sample LUK-4.

Entry	Sample	SSA_{BET} [$m^2\ g^{-1}$]	V_{total} [$cm^3\ g^{-1}$]
1	LUK-4	1955	1.121

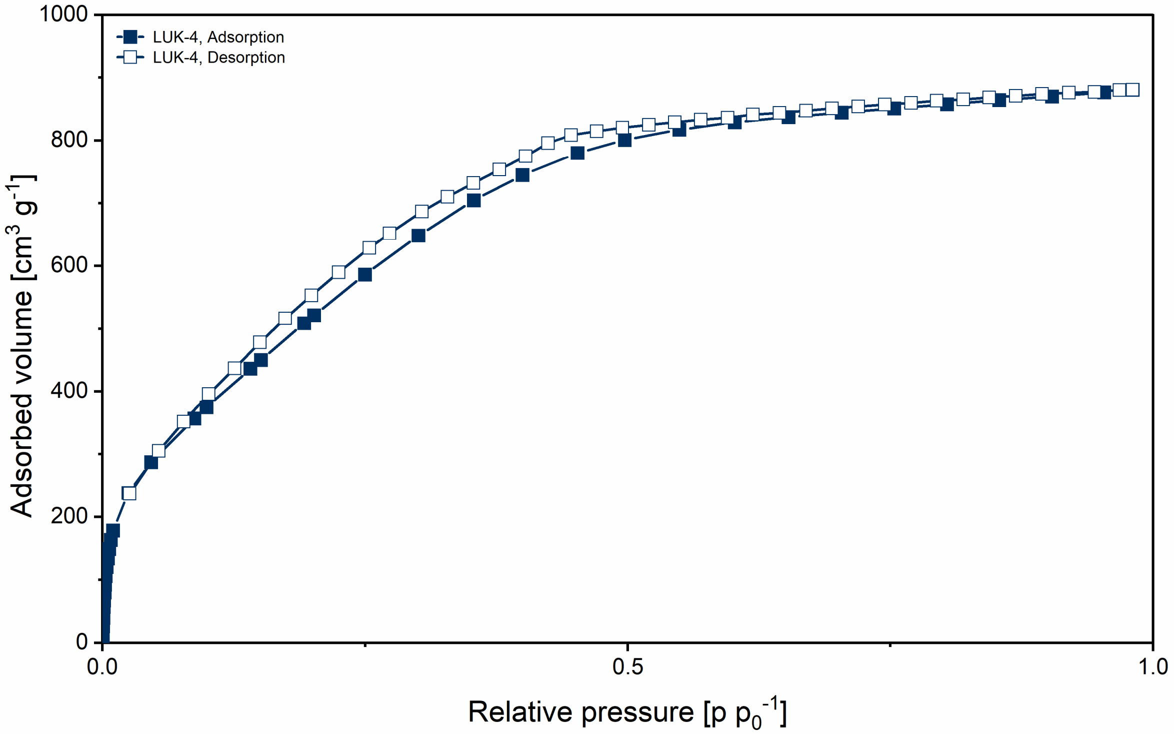

Figure S35. Adsorption and desorption isotherms of LUK-4 taken with Ar-physisorption at 86.7 K.

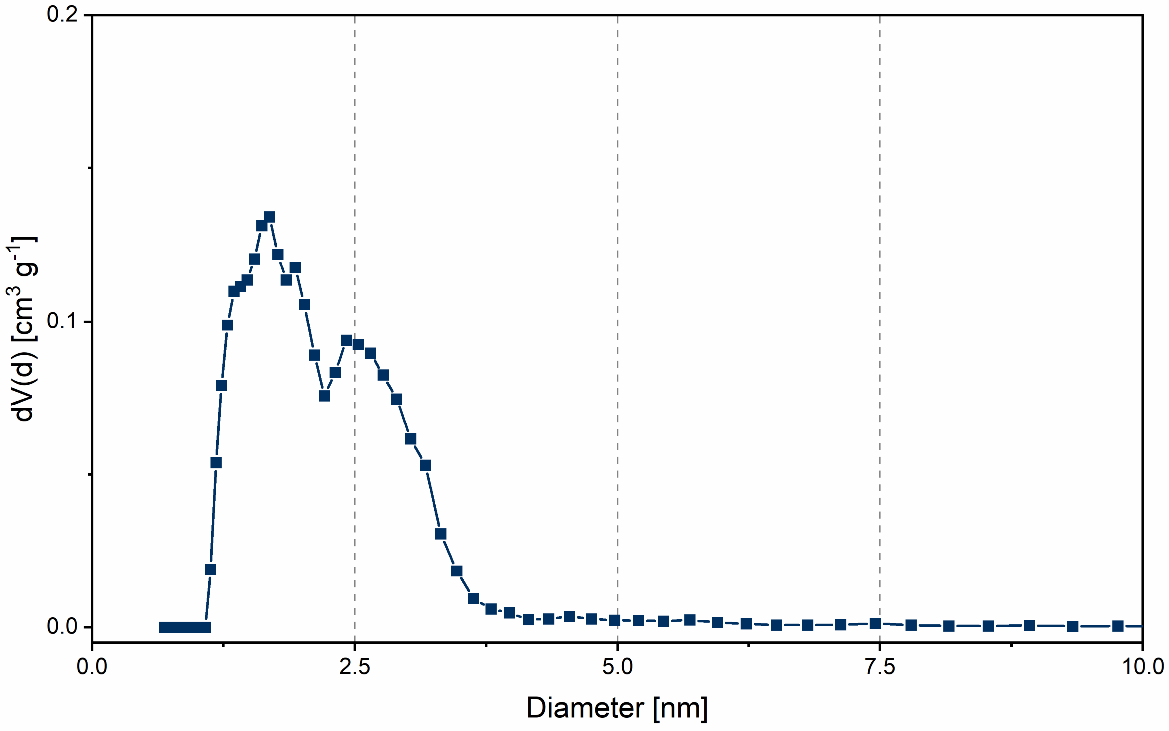

Figure S36. Pore size distribution of LUK-4 calculated from Ar-physisorption via NLDFT method.

Differential scanning calorimetry

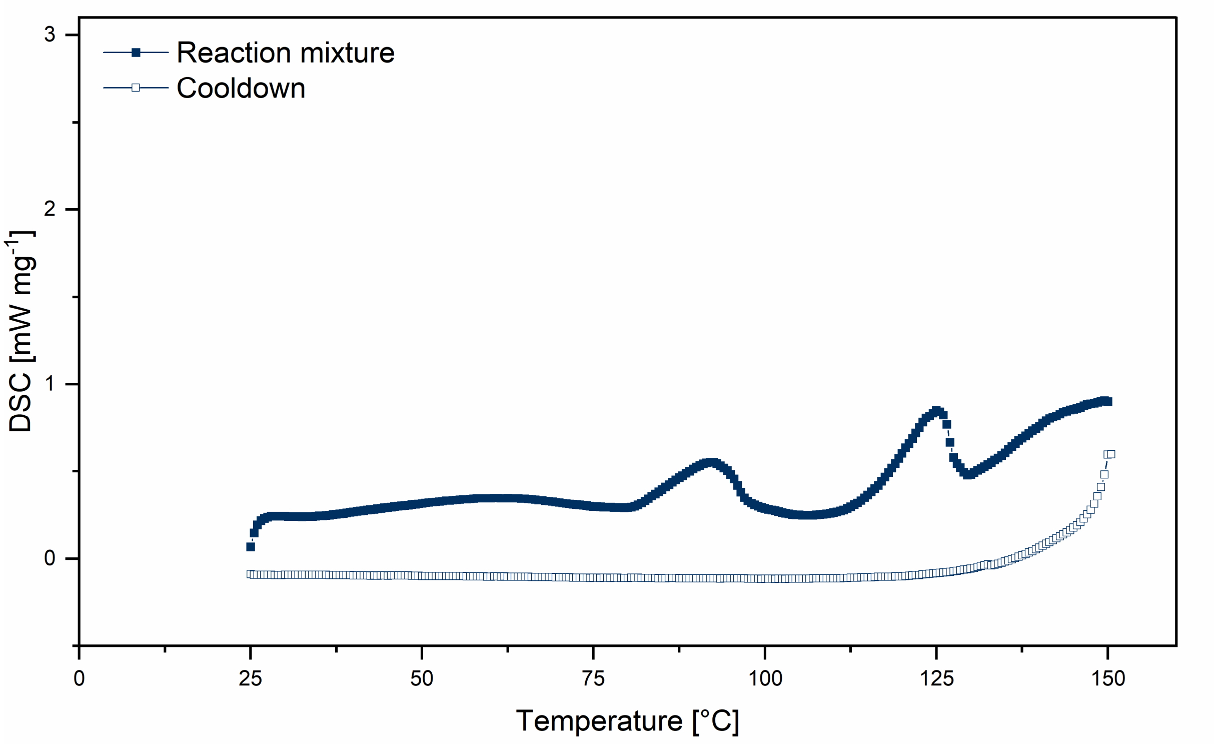

Figure S37. Differential scanning calorimetry measurement of the reaction mixture before extrusion.

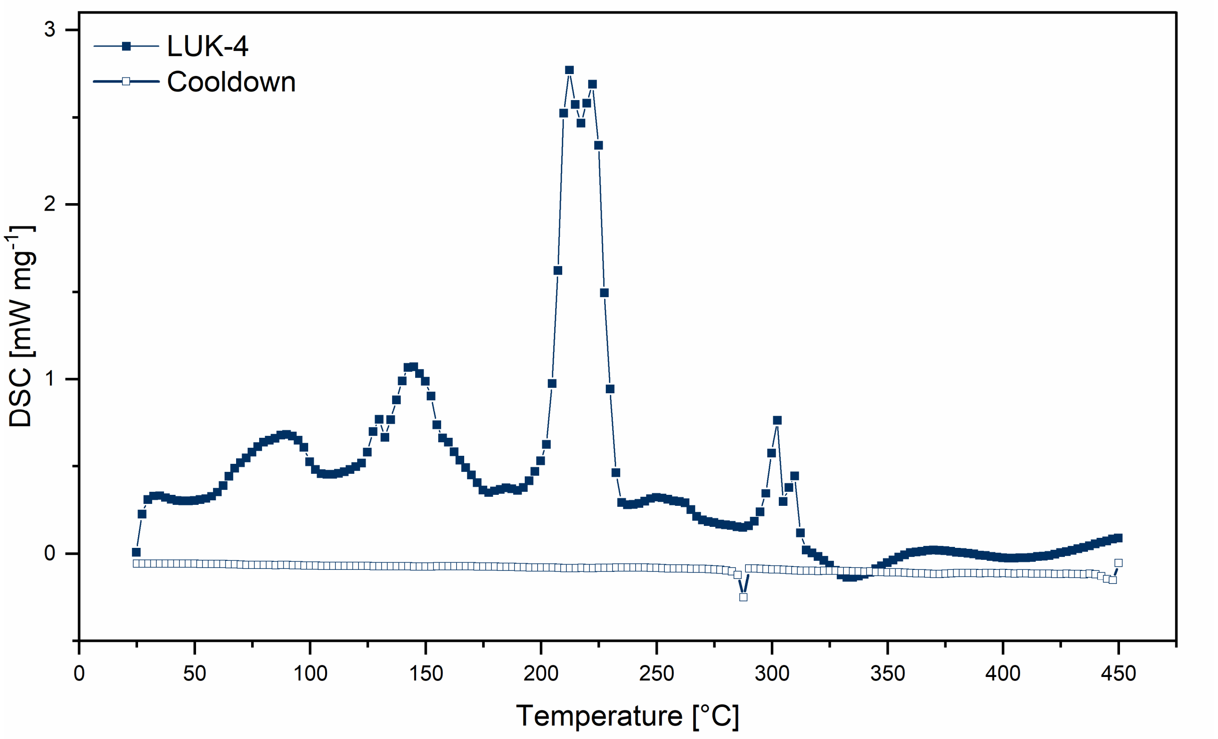

Figure S38. Differential scanning calorimetry measurement of the LUK-4 polymer after extrusion before pyrolysis.

X-ray diffraction

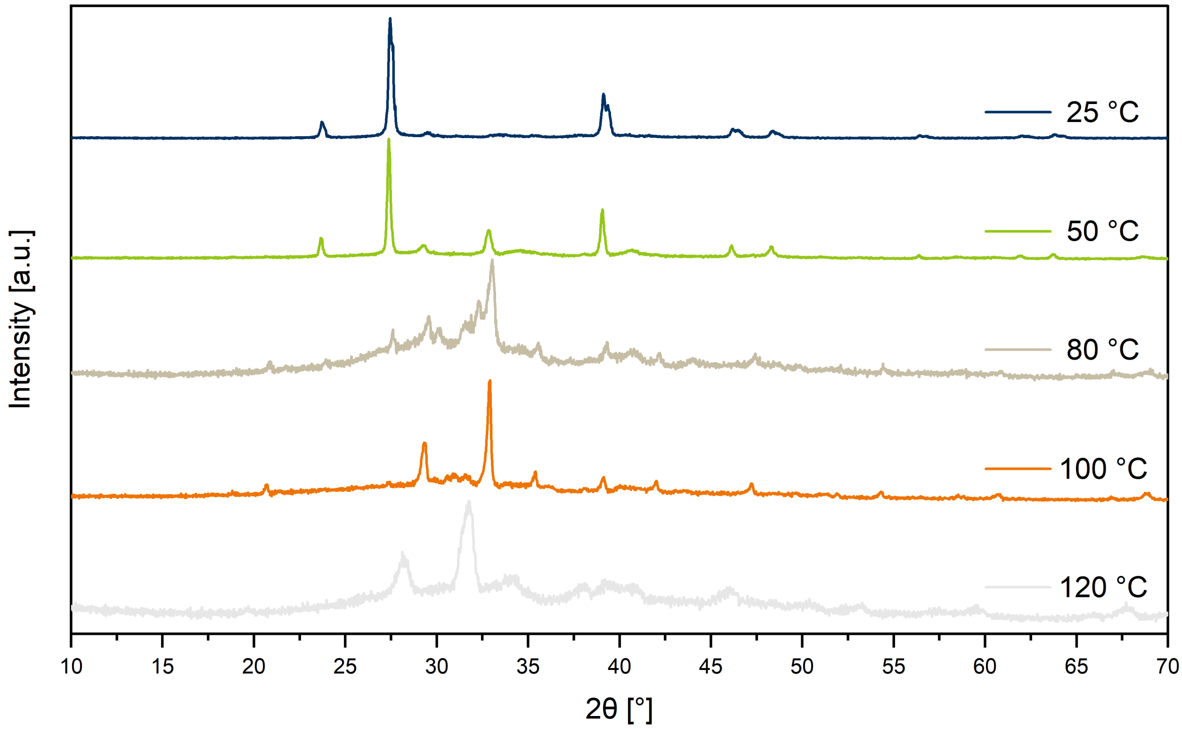

Figure S39. X-ray diffractograms of temperature varied carbon samples after pyrolysis prior washing.

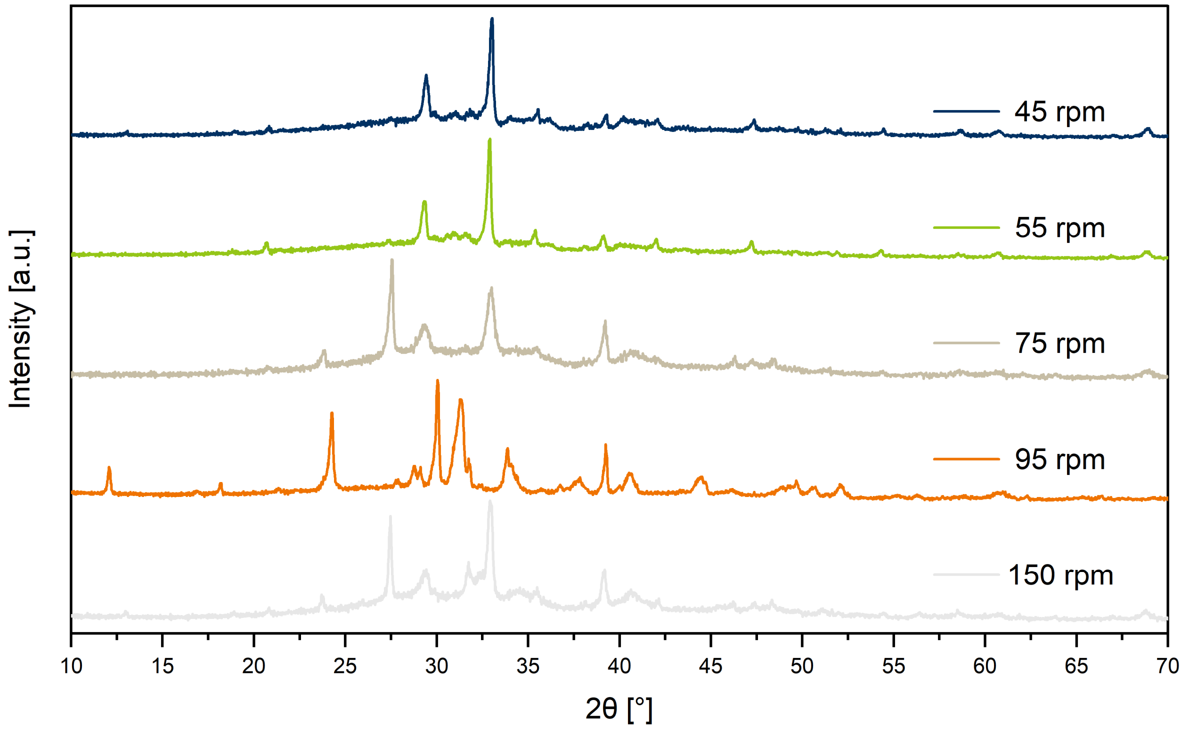

Figure S40. X-ray diffractograms of screw speed varied carbon samples after pyrolysis prior washing.

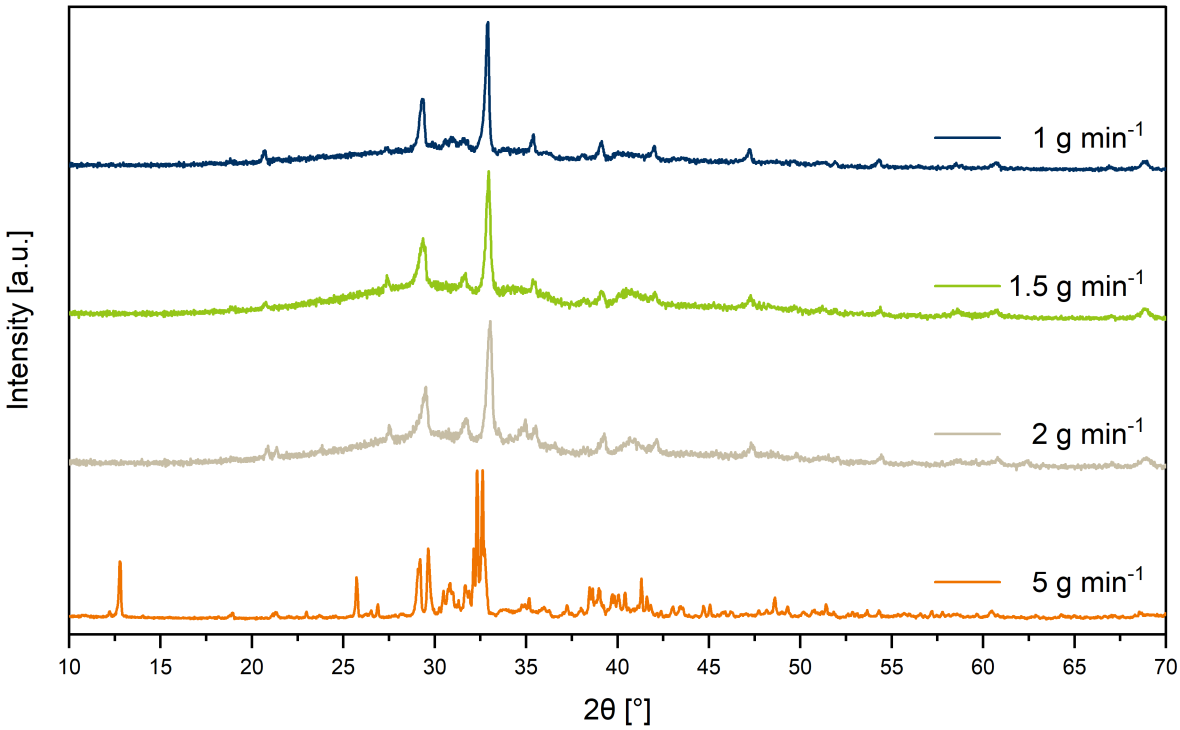

Figure S41. X-ray diffractograms of feed rate varied carbon samples after pyrolysis prior washing.

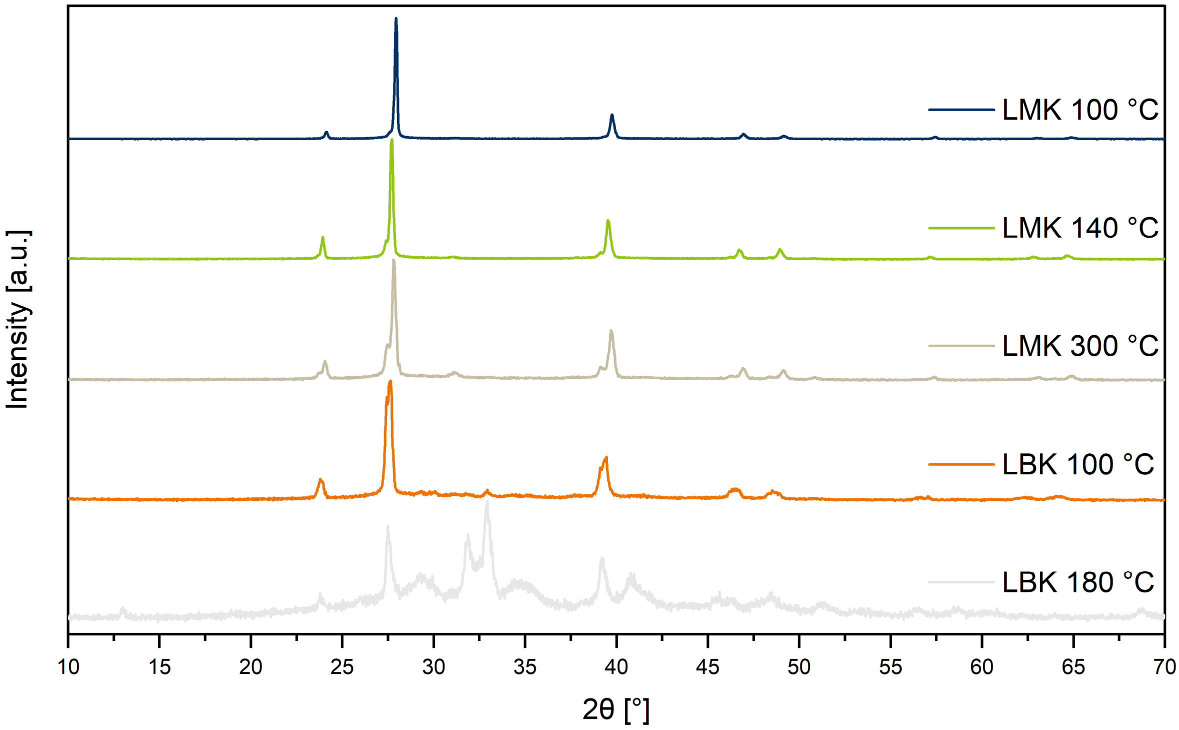

Figure S42. X-ray diffractograms of nitrogen source varied carbon samples after pyrolysis prior washing.

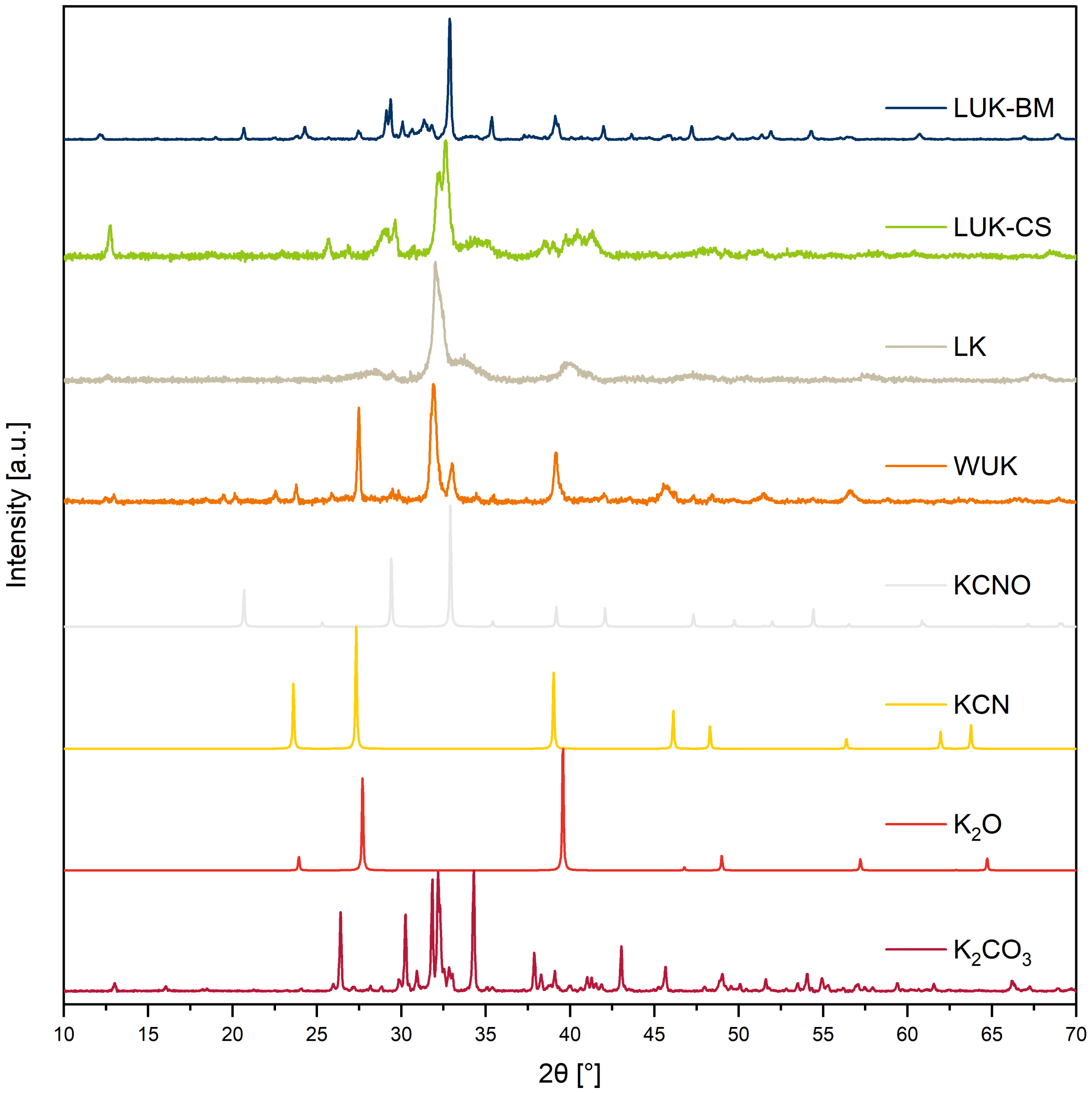

Figure S43. X-ray diffractograms of reference carbon samples after pyrolysis prior washing. X-ray diffractograms of KCNO, KCN and K_2O were calculated from data available in the crystallography open database under COD1010090, COD1541550 and COD9009055 respectively.

Nitrogen physisorption

Table S6: Comparison of specific surface areas (SSA_{BET}) and pore volumes (V_{total}) between samples PAA-1, PI-1 to PI-9.

Entry	Sample	SSA_{BET} [m^2 g^{-1}]	V_{total} [cm^3 g^{-1}]
1	LUK-1	3211	2.598
2	LUK-2	3380	1.956
3	LUK-3	2520	1.258
4	LUK-4	3326	1.783
5	LUK-5	2762	1.389
6	LUK-6	2804	1.427
7	LUK-7	2943	1.564
8	LUK-8	2773	1.389
9	LUK-9	3037	1.607
10	LUK-10	3126	1.459
11	LUK-11	3857	1.954
12	LUK-12	2739	2.122
13	LMK-1	3037	2.005
14	LMK-2	3165	2.201
15	LMK-3	3316	2.298
16	LBK-1	3384	2.468
17	LBK-2	2962	1.33
18	LUK-BM	2575	1.861
19	LUK-CS	2393	1.189
20	LK	584	0.2602
21	WUK	2581	1.295

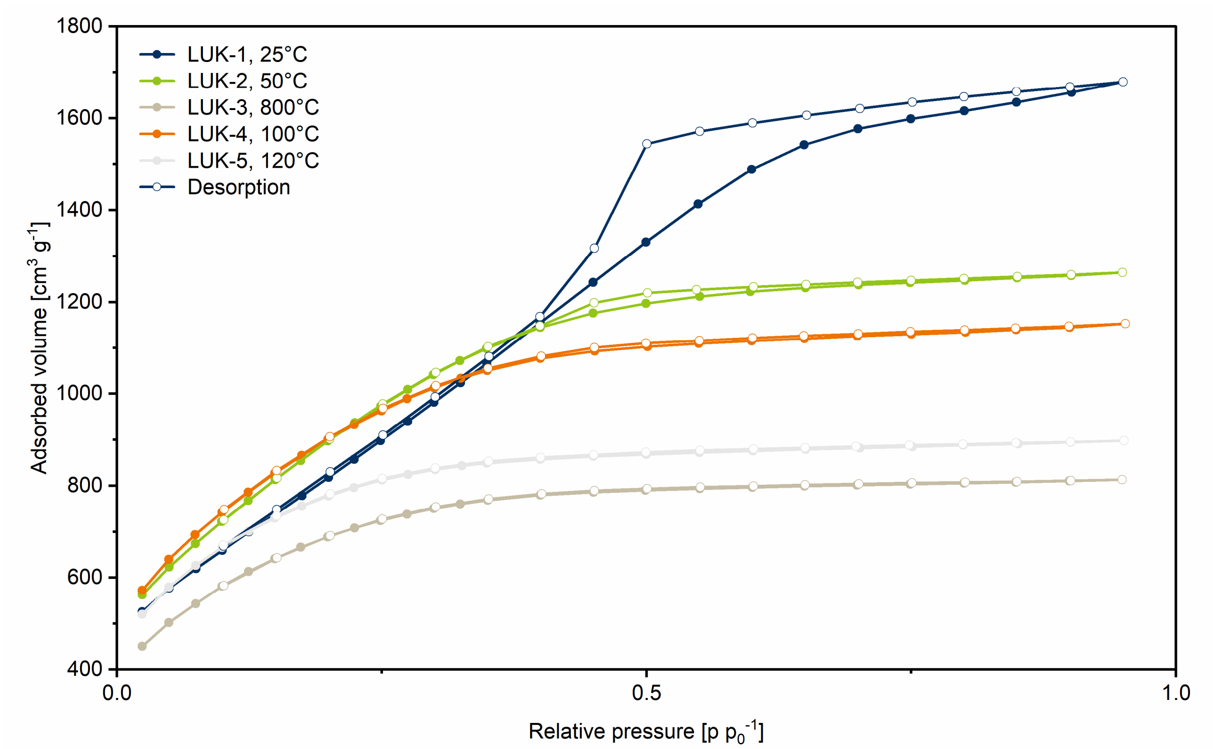

Figure S44. Physisorption isotherms for samples LUK-1, to LUK-5 taken with nitrogen at 78 K.

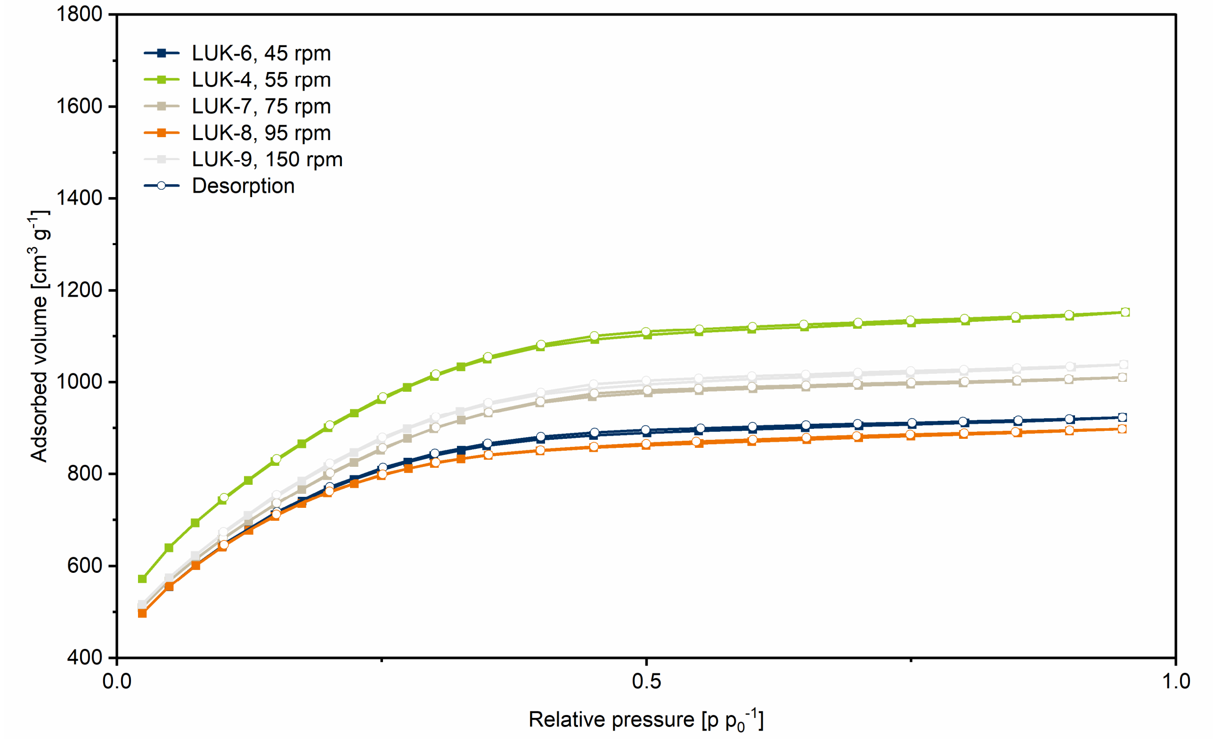

Figure S45. Physisorption isotherms for samples LUK-4, and LUK-6 to LUK-5 taken with nitrogen at 78 K.

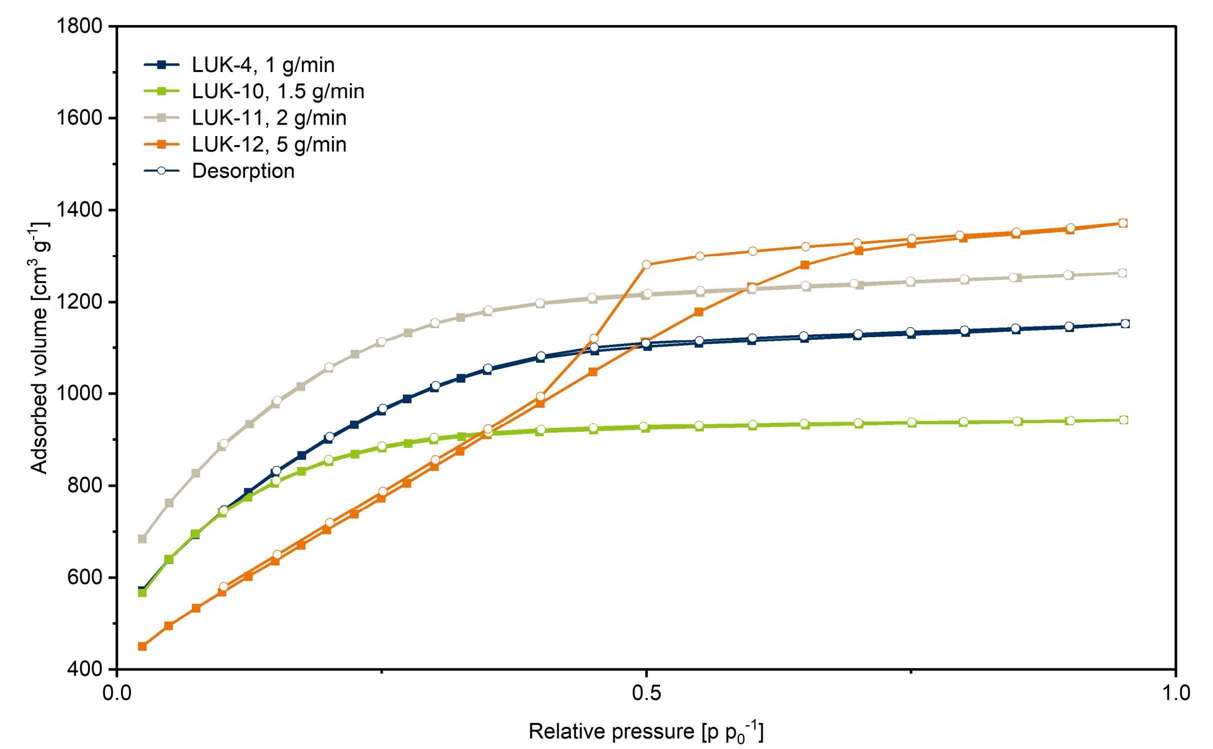

Figure S46. Physisorption isotherms for samples LUK-4, and LUK-10 to LUK-11 taken with nitrogen at 78 K.

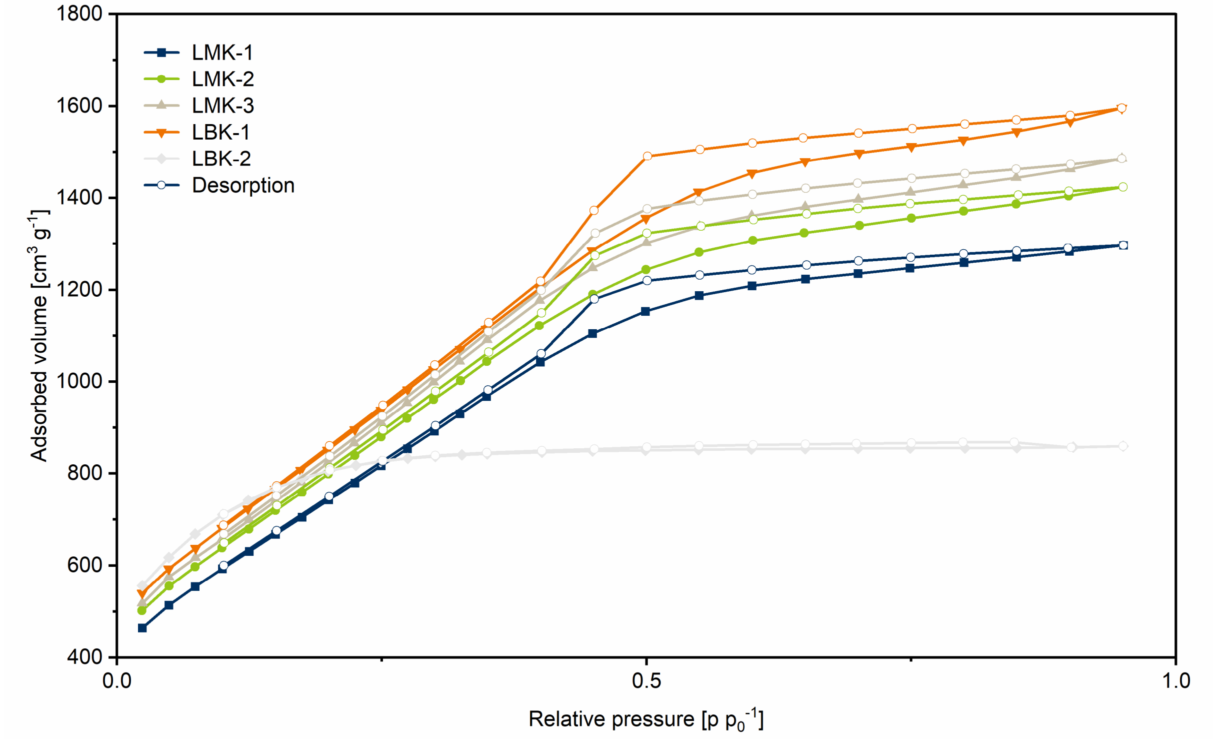

Figure S47. Physisorption isotherms for samples LMK-1 to LMK-3, and LBK-1 and LBK-2 taken with nitrogen at 78 K.

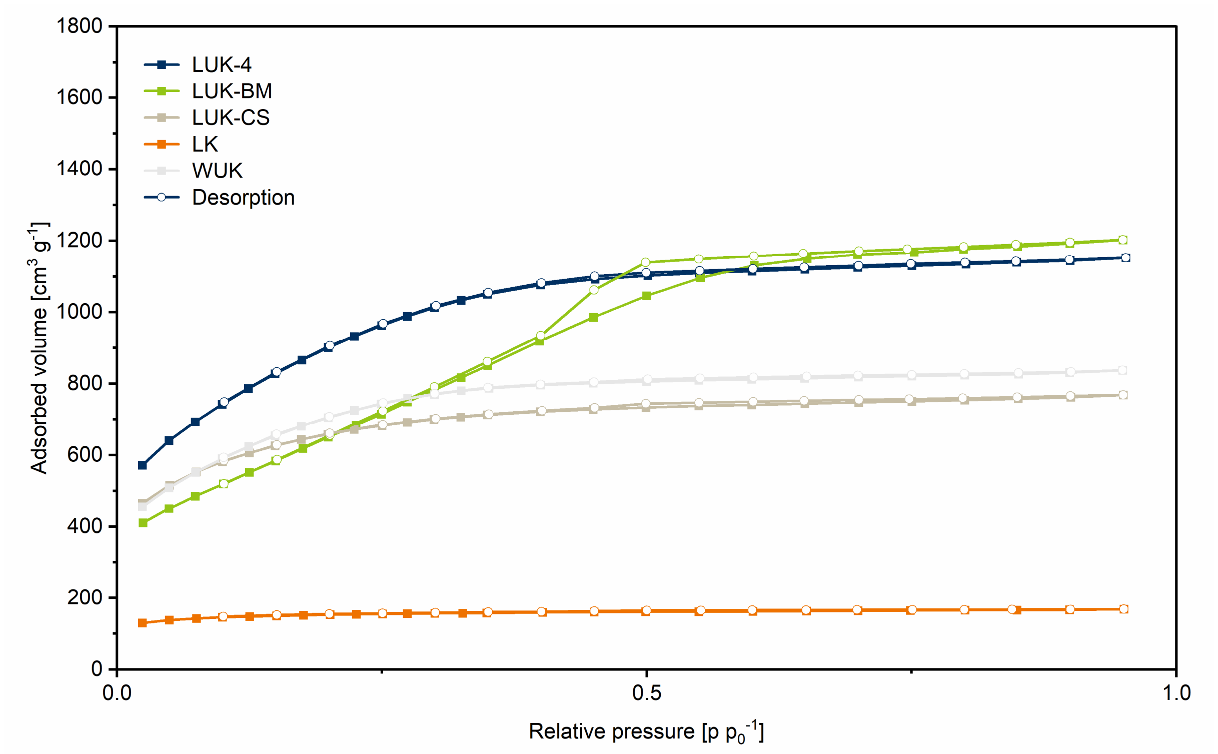

Figure S48. Physisorption isotherms for samples LUK-4, LUK-BM, LUK-CS, LK and WUK taken with nitrogen at 78 K.

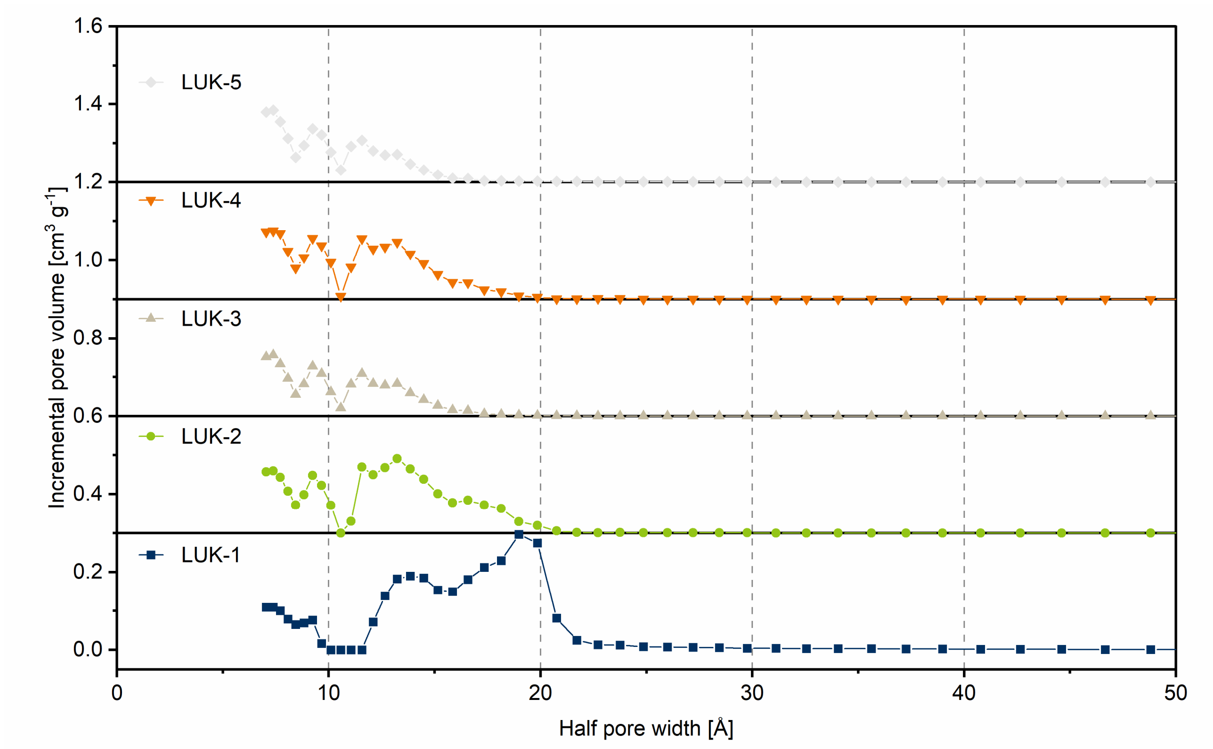

Figure S49. Pore size distribution of temperature varied LUK samples calculated via NLDFT method. Graphs are offset by 0.25 on y-axis.

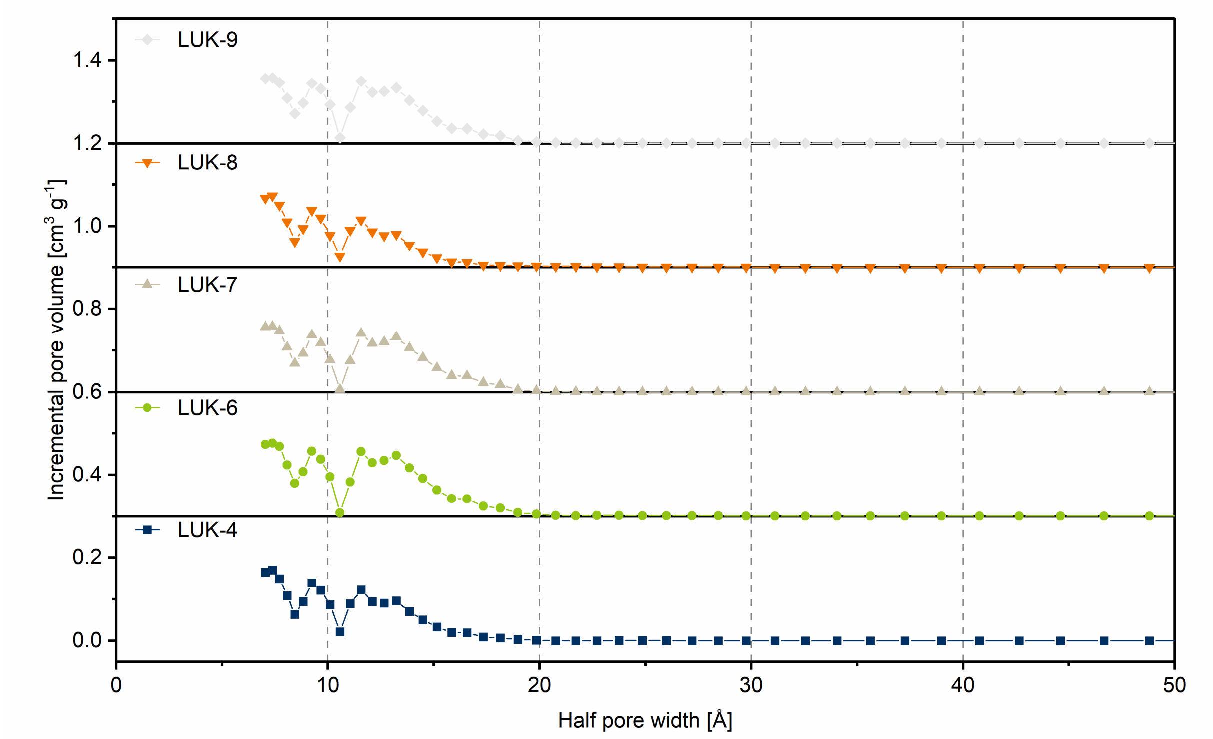

Figure S50. Pore size distribution of rotational speed varied LUK samples calculated via NLDFT method. Graphs are offset by 0.25 on y-axis.

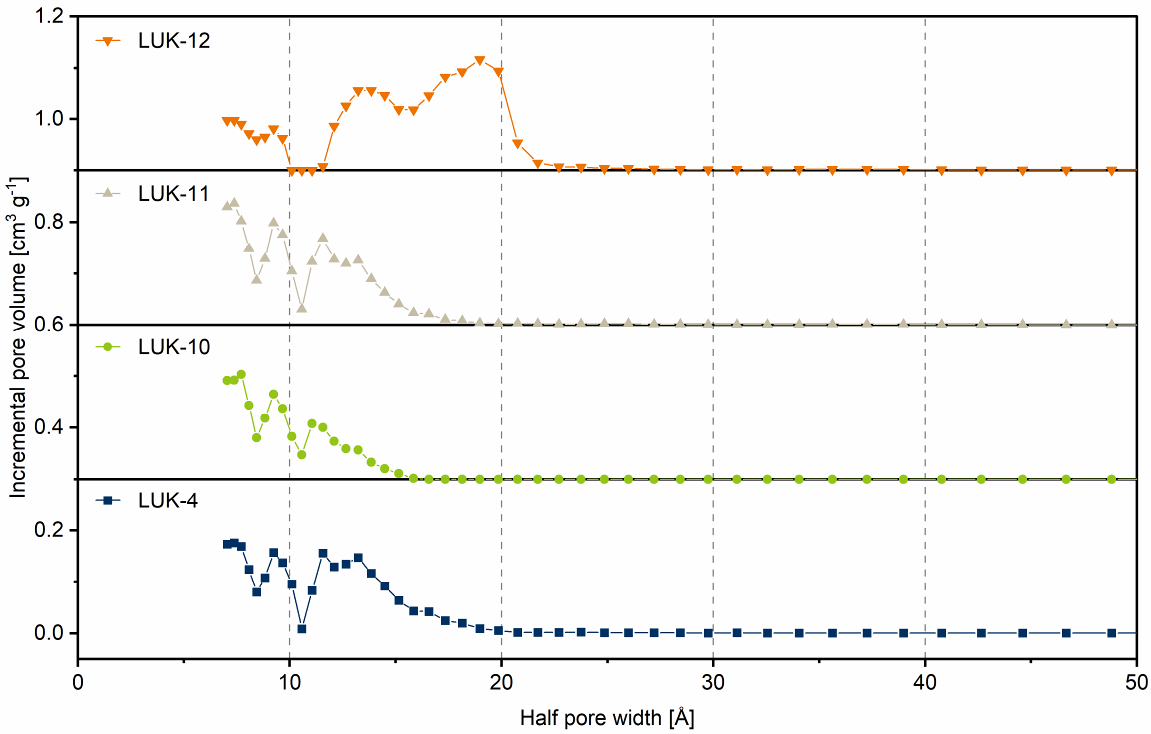

Figure S51. Pore size distribution feed rate varied LUK samples calculated via NLDFT method. Graphs are offset by 0.25 on y-axis.

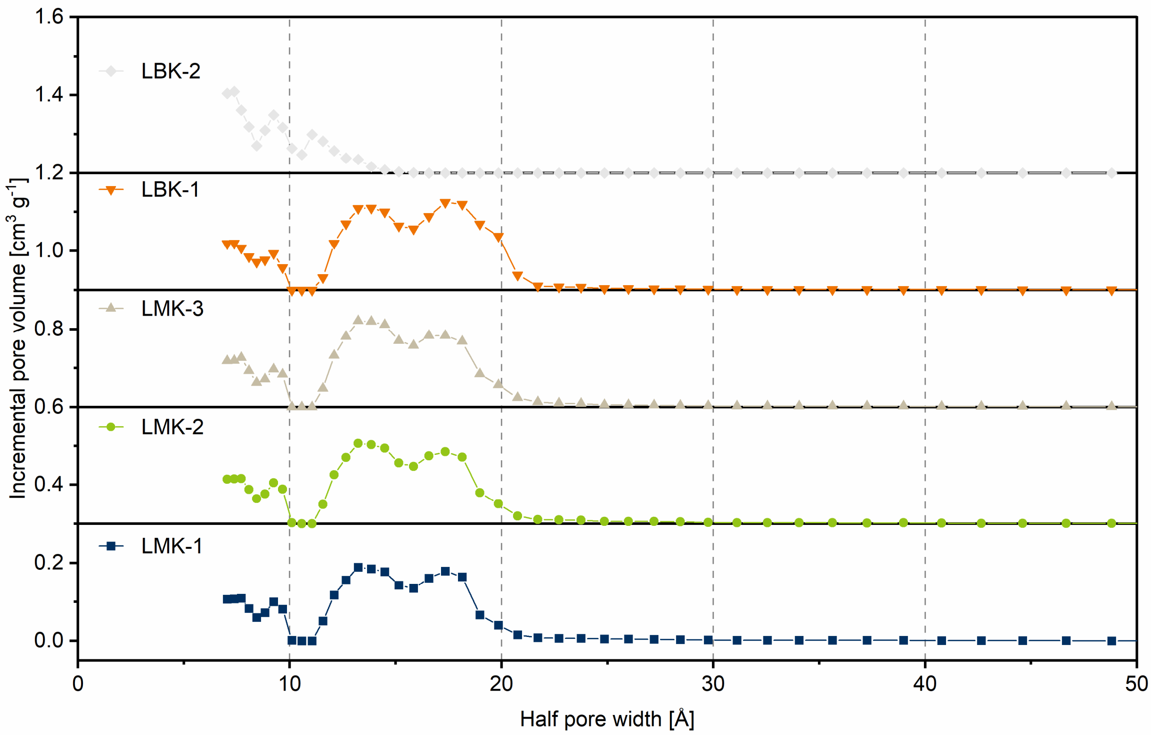

Figure S52. Pore size distribution of nitrogen source varied samples calculated via NLDFT method. Graphs are offset by 0.25 on y-axis.

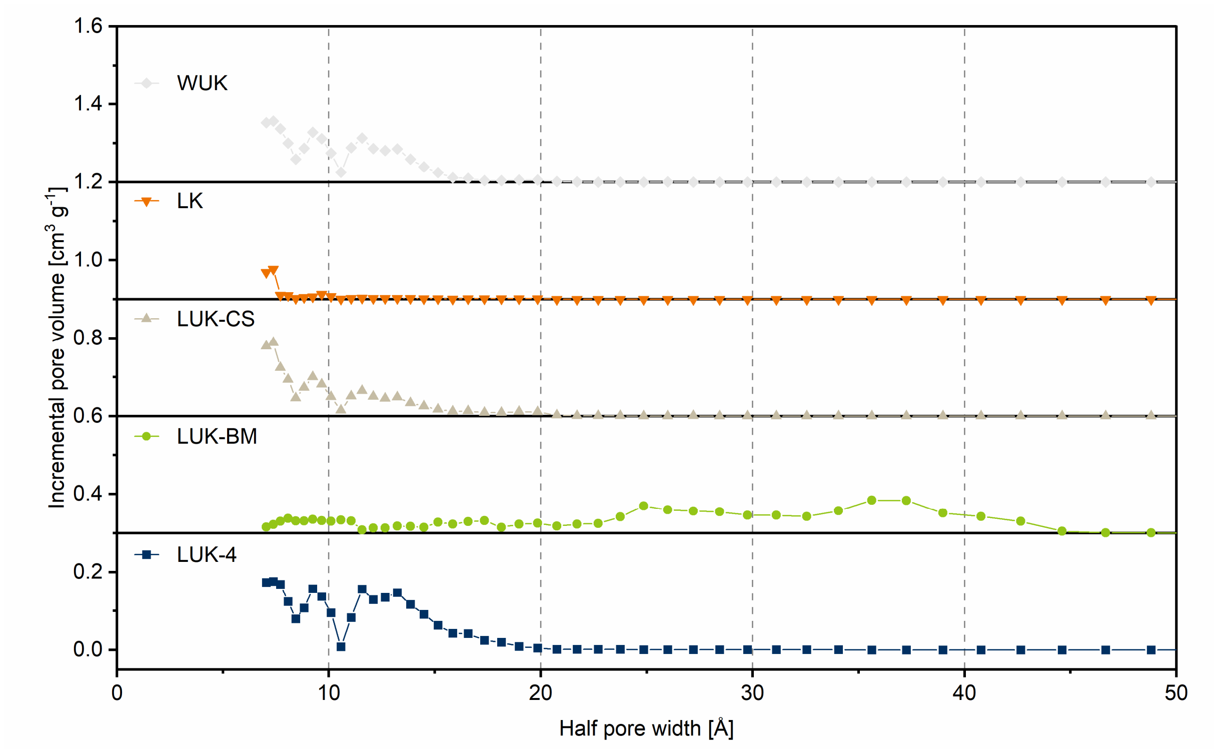

Figure S53. Pore size distribution of reference experiment samples calculated via NLDFT method. Graphs are offset by 0.25 on y-axis.

Space-time yield

Table S7. Space-time yields (STY) of all samples calculated after extrusion, for the composite material and the final purified carbon under assumption of extrusion as a limiting step.

Entry	Sample ID	STY polymer [kg $m^{-3}d^{-1}$]	STY composite [kg $m^{-3}d^{-1}$]	STY carbon [kg $m^{-3}d^{-1}$]
1	LUK-1	42905	16203	1657
2	LUK-2	44569	14455	3070
3	LUK-3	33824	9269	3517
4	LUK-4	30029	10353	3941
5	LUK-5	40510	11869	6277
6	LUK-6	34300	12289	3767
7	LUK-7	38281	12176	4404
8	LUK-8	42427	18917	6270
9	LUK-9	45272	14619	4737
10	LUK-10	50105	19710	8614
11	LUK-11	60506	18811	7554
12	LUK-12	145977	42496	2965
13	LMK-1	31397	12429	1156
14	LMK-2	29871	11715	1245
15	LMK-3	30519	12954	1943
16	LBK-1	24271	9385	1507
17	LBK-2	17061	8265	2411
18	LUK-BM	10746	3968	556
19	LUK-CS	66810	22103	8184
20	LK	42317	23027	8971
21	WUK	41567	9225	3551

Electrochemical analysis

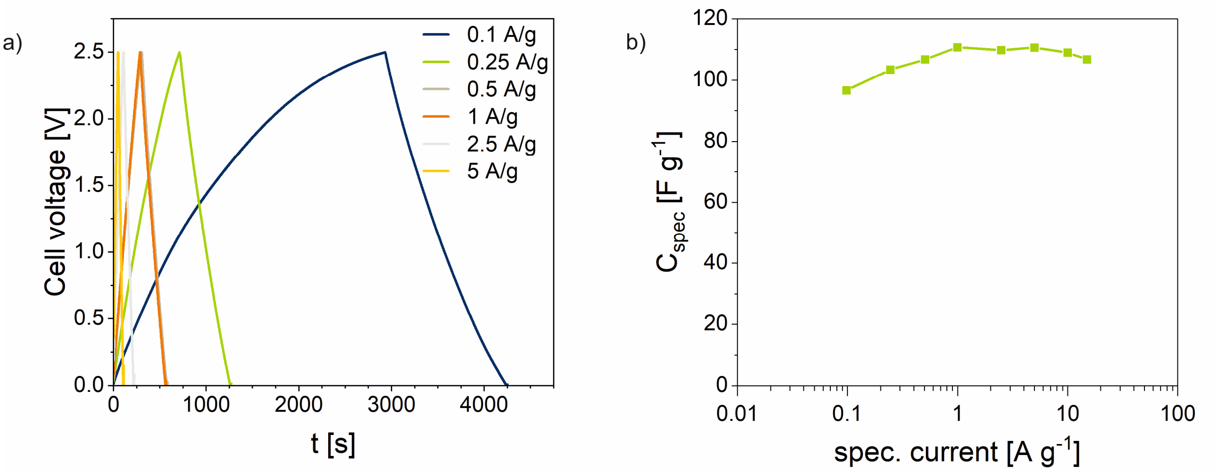

Figure S54. a) Galvanostatic charge and discharge curves (left) and **b)** specific capacitance (right) of a symmetric supercapacitor prepared from LUK-BM with 1M $TEABF_4$/AcN electrolyte.

Table S8. Comparison of electrochemical performance between present work and other supercapacitors based on nanoporous carbons.

Entry	Source	Carbon material	Electrolyte	Specific capacitance [F g^{-1}]
1	Our work	NC from lignin	1 M TEA-BF4 (ACN)	101
2	[1]	NC from lignin	1 M TEA-BF4 (ACN)	147
3	[1]	YP-80F	1 M TEA-BF4 (ACN)	112
4	[2]	AC-W800	1 M TEA-BF4 (ACN)	236
5	[3]	Mesoporous-CDC	1 M TEA-BF4 (ACN)	170
6	[4]	MCNAs	1 M TEA-BF4 (ACN)	152
7	[5]	1700-VAC	1 M TEA-BF4 (ACN)	20
8	[6]	MWCNT	1.5 M TEA-BF4 (ACN)	18

[1] C. Schneidermann, N. Jäckel, S. Oswald, L. Giebeler, V. Presser, L. Borchardt, *ChemSusChem* **2017**, *10*, 2416.

[2] L. Wei, M. Sevilla, A. B. Fuertes, R. Mokaya, G. Yushin, *Adv. Energy Mater.* **2011**, *1*, 356.

[3] Y. Korenblit, M. Rose, E. Kockrick, L. Borchardt, A. Kvit, S. Kaskel, G. Yushin, *ACS nano* **2010**, *4*, 1337.

[4] H.-J. Liu, X.-M. Wang, W.-J. Cui, Y.-Q. Dou, D.-Y. Zhao, Y.-Y. Xia, *J. Mater. Chem.* **2010**, *20*, 4223.

[5] M. Zeiger, N. Jäckel, D. Weingarth, V. Presser, *Carbon* **2015**, *94*, 507.

[6] C. Portet, G. Yushin, Y. Gogotsi, *Carbon* **2007**, *45*, 2511.
